# Characterization of fecal deglucuronidation activity in healthy subjects and in patients treated with fecal microbiota transplantation

**DOI:** 10.1016/j.dmd.2025.100205

**Published:** 2025-11-20

**Authors:** Arttu Uoti, Oona Neulasalmi, Kaisa Hiippala, Timo Oksanen, Perttu Arkkila, Lauri Puustinen, Reetta Satokari, Noora Sjöstedt

**Affiliations:** 1 Drug Research Program, Division of Pharmaceutical Biosciences, Faculty of Pharmacy, University of Helsinki, Helsinki, Finland; 2 Human Microbiome Research Program, Faculty of Medicine, University of Helsinki, Helsinki, Finland; 3 Department of Gastroenterology, Helsinki University Hospital, Helsinki, Finland; 4 Department of Internal Medicine, Faculty of Medicine, University of Helsinki, Helsinki, Finland

**Keywords:** Deglucuronidation, Individual variability, Enterohepatic recycling, Recurrent *Clostridioides difficile* infection, *β*-Glucuronidase

## Abstract

Gut bacterial *β*-glucuronidase (GUS) enzymes contribute to the intestinal toxicity and/or enterohepatic recycling of glucuronidated compounds by cleaving glucuronide conjugates excreted into the intestinal lumen. The activities and substrate specificities of several GUS isoforms have been recently described. However, the extent of intraindividual and interindividual variability in gut microbial deglucuronidation activity has remained poorly characterized. In this study, we used pan-GUS reporter substrates as well as drug and steroid glucuronides to study the deglucuronidation activities of fecal lysates produced from individual fecal samples from healthy donors (*n =* 12), and sequential samples collected from fecal microbiota transplantation (FMT) donors (*n =* 3) and patients with recurrent *Clostridioides difficile* infection who underwent FMT (*n =* 7). To determine relationships between fecal deglucuronidation activity and gut microbiota composition, we used 16S rRNA gene sequencing to characterize the healthy donors’ fecal microbiotas. Although we observed considerable interindividual variability specifically in the processing of steroid glucuronides, intraindividual variability in the fecal deglucuronidation activity of FMT donors was relatively modest. We observed the female sex and *Alistipes*, *Faecalibacterium*, and *Gemmiger* taxa to be associated with higher deglucuronidation activity, whereas the abundance of *Roseburia* correlated negatively with deglucuronidation activity. In addition, the baseline deglucuronidation activity of patients with recurrent *C. difficile* infection was low but increased by FMT treatment. The results of this study further highlight deglucuronidation as a function of a healthy gut microbiota. Moreover, these results improve our understanding of deglucuronidation activity as a source of individual variability in the pharmacokinetics and pharmacodynamics of glucuronidated drugs that undergo enterohepatic recycling.

**Significance Statement:**

Gut microbial deglucuronidation rates of specific compounds may vary considerably between individuals. Deglucuronidation activity is relatively stable within healthy individuals for ≥1 year, but fecal microbiota transplantation can significantly alter the deglucuronidation activity of an individual.

## 1 Introduction

Individual variability in pharmacokinetic processes can compromise drug efficacy and safety. Besides the metabolic enzymes and transporter proteins expressed in the human liver and intestine, the metabolic activity of the gut microbiota may also contribute to pharmacokinetic and pharmacodynamic variability.^1^^,^^2^ Of note, gut microbial *β*-glucuronidase (GUS) enzymes, which deconjugate glucuronide metabolites excreted into the intestinal lumen, play a key role in the enterohepatic recycling (EHR) of glucuronidated drugs.^3^ Pharmacokinetic and pharmacodynamic variability associated with drugs that undergo EHR could thus in part result from variability in the gut microbiota’s deglucuronidation activity.^4^^,^^5^

Previous studies have established GUS expression and activity to be characteristics of a healthy gut microbiota. GUS enzymes are likely important regulators of the homeostasis of many endobiotic substances, such as the hormones and neurotransmitters excreted as glucuronide metabolites to the intestine.^6^, ^7^, ^8^ However, analyses of individual fecal samples have revealed the number of GUS genes to vary between individuals,^8^, ^9^, ^10^, ^11^ and individual GUS enzymes have been shown to exhibit clear substrate specificities and considerable variability in their glucuronide processing rates.^6^^,^^8^, ^9^, ^10^^,^^12^, ^13^, ^14^, ^15^, ^16^ Therefore, it is plausible that individual gut microbiotas would also possess greatly differential drug glucuronide processing capabilities. Indeed, previous studies on fecal microbiota–derived material have revealed considerable variability in the deglucuronidation rates of select drug and endobiotic glucuronides,^8^^,^^10^^,^^11^^,^^17^^,^^18^ and the magnitude of this variability appears to be substrate dependent. However, because previous research has largely focused on individual GUS enzymes instead of fecal microbiota–derived material, relatively little is still known about the extent of interindividual variability in drug glucuronide processing by the fecal microbiota.

Although the overall composition of the gut microbiota of healthy individuals is relatively stable over time,^19^ the use of antibiotics and other drugs,^20^ gastrointestinal diseases,^21^ and diet,^22^ for example, have been associated with changes in the composition of the human gut microbiota. Consequently, GUS expression and gut microbial deglucuronidation activity could also be subject to intraindividual variability. Although a single fecal sample has been estimated to accurately represent an individual’s deglucuronidation activity over a period of 1 to 2 weeks,^23^ longer observation periods are needed to determine the long-term stability of gut microbial deglucuronidation activity at the individual level. Moreover, the gut microbiota can undergo rapid and significant compositional changes, for example, as a result of fecal microbiota transplantation (FMT), which is currently used to treat recurrent *Clostridioides difficile* infection (rCDI) as well as being investigated as a novel treatment for a variety of other gastrointestinal and nongastrointestinal diseases.^24^ To our knowledge, the impact of FMT on deglucuronidation activity and EHR has not been previously studied.

The aim of this study was to extend the current understanding of the individual variability in gut microbial deglucuronidation activity and drug glucuronide processing. To this end, we combined fecal samples and commensal bacterial isolates, a panel of GUS substrates, and microbiota profiling to characterize the fecal deglucuronidation activity of healthy subjects and FMT-treated patients with rCDI. First, we evaluated the interindividual variability in fecal deglucuronidation activity with commonly used pan-GUS reporter substrates^8^^,^^12^^,^^13^ and then with clinically relevant glucuronide conjugates of drugs and endobiotics, namely, ezetimibe, indomethacin, telmisartan, estradiol, and testosterone. These glucuronides are known substrates of hepatobiliary transporters and thus expected to be excreted into the intestinal lumen and/or be involved in EHR.^25^, ^26^, ^27^, ^28^, ^29^ To understand relationships between the gut microbiota composition and variability in deglucuronidation activity, we used 16S rRNA gene sequencing for fecal microbiota profiling. In addition, we studied the intraindividual variability in fecal deglucuronidation activity with longitudinal fecal samples collected from FMT donors and patients with rCDI treated with FMT. The results of this study indicate gut microbial deglucuronidation activity as a source of individual variability in drug pharmacokinetics, particularly for drugs that are glucuronidated and undergo EHR. Furthermore, our results increase the understanding of the effects of FMT on gut microbial deglucuronidation activity.

## 2 Materials and methods

### 2.1 Materials

The GUS reporter substrates 4-methylumbelliferyl-*β*-d-glucuronide dihydrate (4-MUG) and *p*-nitrophenyl-*β*-d-glucuronide (PNPG) were obtained from Santa Cruz Biotechnology and Sigma-Aldrich, respectively, whereas the aglycones 4-methylumbelliferone (4-MU) and *p*-nitrophenol (PNP) were both from Sigma-Aldrich. (*R,S*)-Ezetimibe phenoxy-*β*-d-glucuronide (ezetimibe-G), indomethacin acyl-*β*-d-glucuronide (indomethacin-G), and telmisartan acyl-*β*-d-glucuronide (telmisartan-G) were purchased from Synthose. Ezetimibe was purchased from Sigma-Aldrich and telmisartan from Santa Cruz Biotechnology. Indomethacin was obtained from Orion Pharma. *β*-Estradiol 17-*β*-d-glucuronide (estradiol-17G), estradiol, and testosterone were from Sigma-Aldrich, and testosterone *β*-d-glucuronide (testosterone-G) was from Sigma-Aldrich and National Analytical Reference Laboratory. Aqueous glycerol solution of GUS from *Escherichia coli* and Roche cOmplete EDTA-free Protease Inhibitor Cocktail tablets were purchased from Sigma-Aldrich, and the Pierce Bradford Protein Assay kit was from Thermo Fisher Scientific. All other chemicals and solvents were from Sigma-Aldrich unless stated otherwise. All water was purified with a Milli-Q purification system equipped with a 0.22-*μ*m filter (Merck Millipore).

### 2.2 Research subjects

Interindividual variability in fecal deglucuronidation activity was studied with fecal samples collected from 12 adults (7 female and 5 male, defined as sex assigned at birth) in self-reported good state of health from Uusimaa, Finland (donors HD01–HD12). Characteristics of the study subjects are presented in Supplemental Table 1. Exclusion criteria for participation were diagnosed intestinal disease (eg, inflammatory bowel disease or celiac disease), chronic infection (eg, hepatitis or HIV), and oral or intravenous antibiotic use within the past 6 months. Donors with nonsymptomatic irritable bowel syndrome at the time of sample donation were allowed to participate; in addition, the use of medicines other than antibiotics was not restricted before sample donation. The fecal samples were collected between May 2023 and September 2024 by the study subjects, stored at 4 °C if necessary, and frozen as aliquots at –80 °C within 24 hours after collection. The collection and analysis of the samples were approved by the ethics committee at Helsinki University Hospital (HUS/12203/2022), and all participants gave their informed consent for participation.

Intraindividual variability in fecal deglucuronidation activity was studied with sequential fecal samples collected from 3 study subjects (here donors FD01–FD03), who acted as FMT donors in a previous study.^30^ In addition, fecal samples from 7 patients (here P01–P07) with rCDI were collected before and after FMT during a 1-year follow-up as a part of the previous study^30^ and used to assess fecal deglucuronidation activity before and after FMT. FMT resolved rCDI in all patients, who remained asymptomatic and were not treated with antibiotics during the follow-up period. The samples, which were collected between May 2012 and March 2014, were stored at –80 °C before use in this study. Sample collection and analysis was approved by the ethics committee at Helsinki University Hospital (HUS 124/13/03/01/11 and HUS/1405/2020), and informed written consent was obtained from all participants.

### 2.3 Fecal lysate preparation

Fecal lysates were prepared as described by Jariwala et al^31^ with minor modifications. First, approximately 1 g of thawed feces was suspended in 5 mL of ice-cold extraction buffer (25 mM 4-(2-hydroxyethyl)-1-piperazineethanesulfonic acid, 25 mM NaCl, pH 6.5) supplemented with Roche cOmplete EDTA-free Protease Inhibitor Cocktail and vortexed at full speed with 5 autoclaved silica beads (*d* = 3 mm) for 2.5 minutes. The fecal suspension was then centrifuged at 300*g* and +4 °C for 5 minutes, after which the supernatant was collected, and the extraction repeated for the fecal pellet. The supernatants were then combined and centrifuged again at 300*g* and +4 °C for 5 minutes. The final supernatant was then collected and ultrasonicated with the Branson Sonifier 450 Analog Cell Disruptor (Branson Ultrasonics) with a tapered microtip (*d* = 3 mm) attachment. Each sample was sonicated at 0.5-second pulses and half-maximal ultrasonic intensity for 10 seconds, followed by a 20-second incubation on ice for a total of 8 cycles. The resulting lysate was then centrifuged at 16,000*g* and +4 °C for 25 minutes. Small-molecule contaminants were removed with ultrafiltration through the Amicon Ultra-15 30 kDa centrifugal filter device (Merck Millipore) by centrifugation at 3200–5000*g* and +4 °C for 15–50 minutes, followed by 3 washes with fresh extraction buffer. After the collection of the concentrated fecal lysate, the total protein concentration was determined with the Pierce Bradford Protein Assay kit (Thermo Fisher Scientific) using bovine serum albumin as a reference standard. The lysates were diluted with extraction buffer to 0.25 or 0.5 mg/mL, snap-frozen with liquid nitrogen, and stored at –80 °C until use. For studies with pooled fecal lysate, the lysates from donors HD01–HD05 were combined at equal parts, whereas heat-inactivated lysates to act as controls for spontaneous glucuronide cleavage were produced by incubating the lysates at ≥+70 °C for 10 minutes.

### 2.4 Bacterial isolate lysate preparation

Bacterial isolates originating from fecal samples of healthy donors^32^, ^33^, ^34^, ^35^ were cultured in agar plates or 2–4 mL of appropriate broth for 24–72 hours (Supplemental Table 2), collected by centrifugation at 10,000 rpm for 3 minutes, and washed with phosphate-buffered saline before freezing and storage at –80 °C. For lysate preparation, the bacterial pellets were first suspended in 4 or 8 mL of extraction buffer supplemented with Roche cOmplete EDTA-free Protease Inhibitor Cocktail. The lysates were then prepared as described in the section Fecal lysate preparation. After centrifugation of the bacterial lysates, the supernatants were collected and diluted to 0.5 mg/mL if necessary, snap-frozen with liquid nitrogen, and stored at –80 °C until use.

### 2.5 Deglucuronidation assays

The deglucuronidation assays with 4-MUG and PNPG as substrates were performed on standard clear polystyrene 96-well plates (Sarstedt) at a final reaction volume of 100 *μ*L. First, fecal or bacterial isolate lysates were added to the well plate kept on ice, and the substrates were diluted in reaction buffer (25 mM 4-(2-hydroxyethyl)-1-piperazineethanesulfonic acid, 25 mM NaCl, pH 6.5). The lysates and substrate solutions were then preincubated at +37 °C for 10 minutes, after which the reaction was initiated by the addition of the substrate solution to the lysate. The addition of reaction buffer without substrate acted as the blank fluorescence or absorbance control. In the fecal lysate studies, the final assay concentrations were 0.1 mg/mL total protein for the fecal lysate, 200 *μ*M and 500 *μ*M for 4-MUG and PNPG, respectively, and a maximum of 0.5% for DMSO. In the bacterial isolate studies, the total protein concentration was 0.01 mg/mL or 0.1 mg/mL, and 4-MUG was used both at 200 *μ*M and 250 *μ*M, while other assay parameters were the same as in fecal lysate studies. Aglycone formation was monitored continuously at +37 °C with the Varioskan LUX Multimode Microplate Reader (Thermo Fisher Scientific). Formation of 4-MU was monitored with fluorescence detection (*λ*_excitation_ = 330 nm, *λ*_emission_ = 450 nm), whereas PNP formation was monitored by measuring absorbance at 400 nm. For the quantification of the released substrate, 4-MU and PNP standard solutions were prepared fresh for each experiment. The range of concentrations was typically between 0.25 and 50 *μ*M for 4-MU and 5 and 250 *μ*M for PNP.

The deglucuronidation of drug glucuronides was studied essentially as previously described.^17^ Briefly, fecal or bacterial isolate lysates or the *E. coli* GUS was first added to a conical-bottom 96-well plate (Greiner Bio-One) kept on ice, and the substrates were then diluted in reaction buffer. After preincubation of the lysates and substrate solutions at +37 °C for 10 minutes, the reaction was initiated by adding the substrate solution to the lysate, followed by brief shaking at 500 rpm. The addition of substrate solution to heat-inactivated lysate acted as a control for spontaneous deglucuronidation, whereas lysates treated with only reaction buffer acted as the blank control. The final assay concentrations were 0.1 mg/mL total protein for the fecal or bacterial isolate lysate, 100 *μ*M or 200 *μ*M for the substrate, and a maximum of 0.5% for DMSO, in a final reaction volume of 50 *μ*L. After the desired incubation time, the reaction was stopped by the addition of 100 *μ*L of ice-cold acetonitrile. The samples were then vortexed at full speed for 20 seconds, incubated at room temperature for 10 minutes, and centrifuged at 16,000*g* for 10 minutes. The supernatants were diluted with water, and the released aglycones were quantified with ultraperformance liquid chromatography (UPLC) as described in the section Analytical methods.

### 2.6 Analytical methods

Estradiol, ezetimibe, indomethacin, telmisartan, and testosterone were quantified from the deglucuronidation assay samples with an Acquity UPLC system from Waters coupled with a photodiode array detector. Chromatographic separation of the analytes from estradiol and testosterone samples was achieved with an Acquity UPLC BEH C18 column (1.7 *μ*m, 2.1 × 50 mm; Waters), whereas an Acquity UPLC HSS C18 column (1.8 *μ*m, 2.1 × 50 mm; Waters) was used for the analysis of ezetimibe, indomethacin, and telmisartan samples. The columns were maintained at +30 °C and operated at 0.5 mL/min. The samples were kept at room temperature and injected into the column at 2–5 *μ*L. The chromatographic eluents for all analytes were 15 mM KH_2_PO_4_ at pH 2 (A) and acetonitrile (B). Ezetimibe, indomethacin, and telmisartan were separated with an isocratic elution method with the mobile phase consisting of 50% B for ezetimibe, 55% B for indomethacin, and 35% B for telmisartan samples. Gradient elution was used for estradiol and testosterone samples with the following method: 0–3 minutes 30% → 70% B, 3–3.01 minutes 70% → 30% B, and 3.01–4 minutes 30% B. The retention times of the analytes and the absorbance wavelengths used for analyte quantification were 1.29 minutes and 220 nm for estradiol, 0.84 minutes and 232 nm for ezetimibe, 0.93 minutes and 230 nm for indomethacin, 0.65 minutes and 230 nm for telmisartan, and 1.37 minutes and 245 nm for testosterone, respectively.

### 2.7 DNA extraction and 16S rRNA gene sequencing

DNA was extracted from the fecal samples as described previously by using repeated bead beating as the mechanical cell lysis step.^36^ High-throughput DNA purification was achieved with the KingFisher Flex Purification System (Thermo Fisher Scientific). Amplification of the 16S rRNA gene, indexing, library preparation, and sequencing were conducted by the DNA Sequencing and Genomics Laboratory at the Institute of Biotechnology, University of Helsinki. The V3–V4 region of the 16S rRNA gene was amplified using universal bacterial primers (341F: CCTACGGGNGGCWGCAG, 785R: GACTACHVGGGTATCTAATCC). The DNA extraction and polymerase chain reaction (PCR) amplification protocols included blank samples for the assessment of contamination. The purified PCR products were sequenced with Illumina MiSeq (2 × 300 bp paired-end reads).

### 2.8 Data analysis and statistical testing

All deglucuronidation assays were performed with triplicate samples. Deglucuronidation rate was defined as the amount of formed aglycone per time per amount of total protein in the reaction (nmol/min per mg). For 4-MUG and PNPG, aglycone formation was monitored continuously at 1-minute intervals, and the deglucuronidation rates were determined at initial velocity conditions (generally ≤10% substrate depletion) with linear regression. For drug glucuronides, appropriate incubation times were determined for each substrate, and the deglucuronidation rates were calculated as the amount of formed aglycone per incubation time per amount of total protein in the reaction. Variability in deglucuronidation rates was measured as the CV (%) and the ratio between the highest and the lowest observed rate (max/min fold) both between and within individuals. The deglucuronidation data were analyzed and visualized with GraphPad Prism version 6.07 (GraphPad Software Inc) and in RStudio (Posit PBC) with R version 4.4.2 (R Foundation for Statistical Computing) using ggplot2 version 3.5.1^37^ to generate figures. Spearman correlation from the R package psych version 2.5.3^38^ was used for correlation analyses and the Mann-Whitney *U* test from base R for comparisons between 2 groups. In addition, the Friedman test with Dunn post hoc test from GraphPad Prism was applied for the comparison of the deglucuronidation activities of patients with rCDI after FMT to their baseline before FMT activity.

The demultiplexed raw 16S rRNA gene sequencing data contained a total of 3,285,525 reads with an average of 273,345 reads per fecal DNA sample (range: 177,992–357,303 reads). Only low numbers of reads were present in the blank controls from the DNA extraction protocol (107 reads) and the 2 PCR cycles (524 and 4757 reads). PCR primers were removed from the 5′ ends of the raw paired-end sequences with Cutadapt version 5.0.^39^ Only those paired-end sequences containing the primers were included in subsequent analyses. Thereafter, the trimmed forward reads were denoised with the DADA2 R package version 1.34.0^40^ as instructed in the DADA2 Pipeline Tutorial (available online at https://benjjneb.github.io/dada2/tutorial.html). After filtering, denoising, and chimera removal, an average of 174,193 (range: 151,085–220,481) reads per fecal DNA sample remained (Supplemental Table 3). Taxonomic assignments up to the species level were achieved using the DADA2-formatted Silva training data version 138.2.^41^ The resulting amplicon sequence variant table was then further analyzed with the R packages phyloseq version 1.50.0^42^ and microbiome version 1.28.0,^43^ and visualized with GraphPad Prism and ggplot2. Details of the microbiota data analysis are presented in Supplemental Material 1. Spearman correlation was used for correlation analyses and the Mann-Whitney *U* test for differential abundance testing, whereas the Kolmogorov-Smirnov test was applied for the analysis of differences in alpha diversities between groups.

In the statistical analyses, the Benjamini-Hochberg correction for multiple comparisons was applied to the obtained *P* values, and the corrected values are presented in the Supplemental Materials. Because of the small sample size, however, unadjusted *P* values are reported in the main text, and unadjusted *P* values <.05 were considered statistically significant.

## 3 Results

### 3.1 Interindividual variability in gut microbial deglucuronidation

**Fig. 1:**
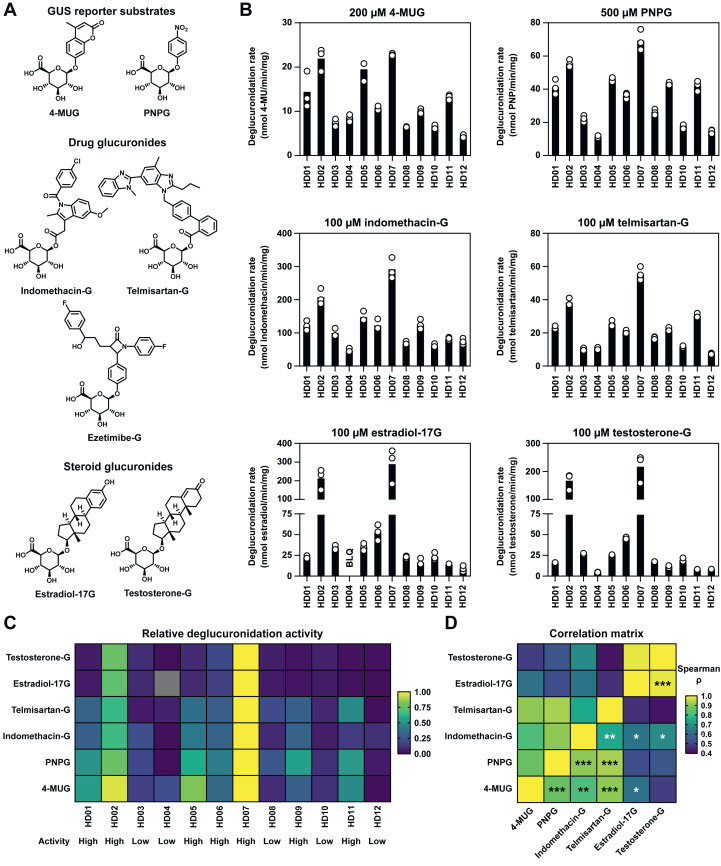
Structures of the GUS reporter substrates, drug glucuronides, and steroid glucuronides investigated in this study (A). Deglucuronidation of 200 *μ*M 4-MUG, 500 *μ*M PNPG, and 100 *μ*M drug and steroid glucuronides in the presence of fecal lysates produced from fecal samples of 12 healthy donors (HD01–HD12) (B). After the initiation of the reaction, 4-MU and *p*-nitrophenol formation in the presence of 10 *μ*g fecal lysates was monitored continuously at 1-minute intervals. The deglucuronidation rates of 4-MUG and PNPG, defined as the amount of formed aglycone per time per amount of total protein in the reaction, were determined with linear regression. The drug and steroid glucuronides were incubated with 5 *μ*g of the fecal lysates for a predetermined time, after which the formed aglycones were measured with ultraperformance liquid chromatography. The incubation times were 1 minute and 3 minutes in all reactions for indomethacin-G and telmisartan-G, respectively. For estradiol-17G, the incubation time was 3 minutes, except for sample HD04 (6 minutes) and samples HD02 and HD07 (1 minute). Similarly, the incubation time was 3 minutes for testosterone-G, except for samples HD02 and HD07 (1 minute). The concentration of formed estradiol for sample HD04 was below the limit of quantification (BLQ). The deglucuronidation data were collected from 3 independent experiments (2 for estradiol-17G and HD04) shown as individual data points representing the deglucuronidation rate of the substrate with the bar representing the mean. Heatmap of the relative deglucuronidation activities of lysates from donors HD01–HD12 (C). Here, the deglucuronidation rate of each substrate was normalized to between 0 and 1 according to the lowest and highest deglucuronidation rates. Spearman correlation matrix of the deglucuronidation rates of the investigated GUS substrates (D). In panels C and D, yellow corresponds to high values and blue to low values. ∗*P* < .05, ∗∗*P* < .01, ∗∗∗*P* < .001. Presented are the unadjusted *P* values; see Supplemental Table 4 for adjusted (Benjamini-Hochberg) values. Estradiol-17G, *β*-estradiol 17-*β*-d-glucuronide; GUS, *β*-glucuronidase; indomethacin-G, indomethacin acyl-*β*-d-glucuronide; 4-MU, 4-methylumbelliferone; 4-MUG, 4-methylumbelliferyl-*β*-d-glucuronide; PNP, *p*-nitrophenol; PNPG, *p*-nitrophenyl-*β*-d-glucuronide; telmisartan-G, telmisartan acyl-*β*-d-glucuronide; testosterone-G, testosterone *β*-d-glucuronide.

Interindividual variability in gut microbial deglucuronidation activity was assessed using fecal lysates produced from fecal samples collected from 12 healthy donors. First, GUS reporter substrates 4-MUG and PNPG (Fig. 1A) were investigated, and both were hydrolyzed by all studied fecal lysates (Fig. 1B). Moreover, interindividual variability in the deglucuronidation rates of the GUS reporters was similar between the 2 substrates (Table 1).

**Table 1:** Measures of interindividual variability of the investigated *β*-glucuronidase substrates in fecal lysates from 12 healthy donors (11 for estradiol-17G, as the estradiol concentrations for HD04 were below the limit of quantification) The individual donors’ deglucuronidation activities are presented in Fig. 1.

Substrate	200 *μ*M 4-MUG	500 *μ*M PNPG	100 *μ*M indomethacin-G	100 *μ*M telmisartan-G	100 *μ*M estradiol-17G	100 *μ*M testosterone-G
Mean (SD) (nmol/min per mg)	12.2 (6.29)	35.5 (17.6)	121 (69.5)	22.8 (13.8)	66.6 (93.3)	47.1 (69.3)
CV (%)	51.7	49.6	57.5	60.5	140	147
Range (nmol/min per mg)	4.48–22.8	11.2–69.3	47.0–292	7.53–55.7	9.06–288	4.79–217
Maximum/minimum fold	5.09	6.17	6.21	7.39	31.8	45.2

estradiol-17G, *β*-estradiol 17-*β*-d-glucuronide; indomethacin-G, indomethacin acyl-*β*-d-glucuronide; 4-MUG, 4-methylumbelliferyl-*β*-d-glucuronide; PNPG, *p*-nitrophenyl-*β*-d-glucuronide; telmisartan-G, telmisartan acyl-*β*-d-glucuronide; testosterone-G, testosterone *β*-d-glucuronide.

We then evaluated the deglucuronidation of drug and steroid glucuronides (Fig. 1A) first at multiple time points using a pooled fecal lysate from 5 donors (Supplemental Fig. 1) and then at a single time point using the 12 individual fecal lysates (Fig. 1B). Indomethacin-G, telmisartan-G, estradiol-17G, and testosterone-G were deglucuronidated by the pooled lysate in a time-dependent manner. Interestingly, however, no ezetimibe was detected when ezetimibe-G was incubated with the pooled lysate for up to 12 minutes or with up to 100 IU of GUS enzyme from *E. coli* for 60 minutes (data not shown), although ezetimibe-G at 100 *μ*M inhibited 4-MUG hydrolysis by pooled fecal lysate by approximately 25% (Supplemental Fig. 2). Therefore, ezetimibe-G was excluded from subsequent analyses. Variability in the deglucuronidation of indomethacin-G and telmisartan-G was comparable between the 2 substrates (Table 1); however, the average rate of indomethacin-G deglucuronidation was approximately 5.3-fold the rate of telmisartan-G deglucuronidation. Moreover, there was considerable variability in the formation of estradiol and testosterone in the presence of the individual fecal lysates; the variability observed in the deglucuronidation of these steroid glucuronides markedly exceeded that of the GUS reporter substrates or the investigated drug glucuronides.

For all substrates, samples from donors HD02 and HD07 exhibited the greatest deglucuronidation rates of all 6 investigated GUS substrates, whereas samples from donors HD04 and HD12 were generally the least active (Fig. 1C). In contrast, while samples from donors HD01, HD05, and HD11 exhibited moderate to high relative deglucuronidation activity (0.5–0.8) of the GUS reporter substrates, their relative deglucuronidation activities of the steroid glucuronides were ≤0.1. The donors were divided into high (*n =* 7) and low (*n =* 5) GUS activity donors using an average relative deglucuronidation activity of 0.25 as an arbitrary cutoff. The Spearman correlation analysis showed a positive correlation between the deglucuronidation rates of all substrates (Fig. 1D; Supplemental Table 4); this correlation was the strongest between the rates of estradiol-17G and testosterone-G deglucuronidation (Spearman *ρ* = 0.982, 95% confidence interval [CI], 0.929–0.995, *P* = 8.40E-08). The poorest observed correlation was between telmisartan-G and the steroid glucuronides.

### 3.2 Deglucuronidation activity of bacterial strains

**Fig. 2:**
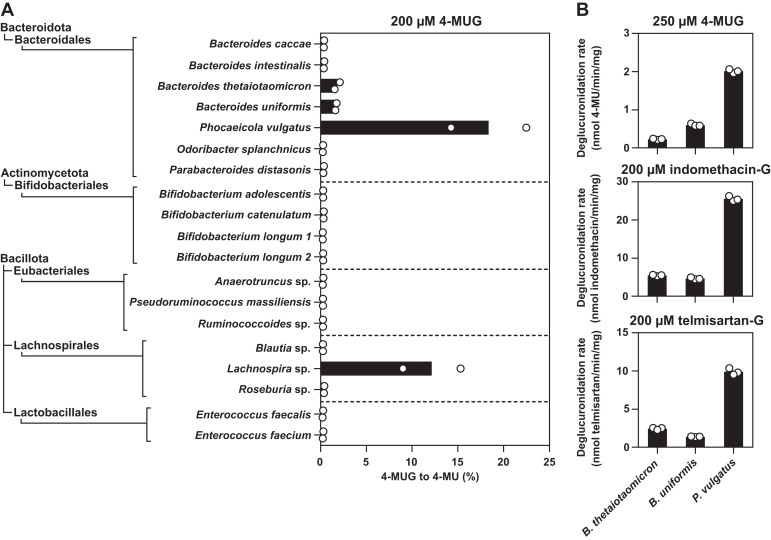
Deglucuronidation of 4-MUG by cultured bacterial strains (A). Lysates (1 *μ*g total protein per reaction) produced from 19 bacterial isolates were incubated with 200 *μ*M 4-MUG for 24 hours at +37 °C with 250 rpm shaking, after which the released 4-MU aglycone was measured using fluorescence detection. The data are presented as the fraction of 4-MUG converted to 4-MU in percentage. The bars represent the mean of 2 individual experiments (shown as points) conducted on 2 separately prepared lysate batches. Deglucuronidation of 250 *μ*M 4-MUG, 200 *μ*M indomethacin-G, and 200 *μ*M telmisartan-G (B) in the presence of Bacteroidales lysates (10 *μ*g total protein for 4-MUG and 5 *μ*g for indomethacin-G and telmisartan-G). For 4-MUG, aglycone formation was monitored continuously at 1-minute intervals. The deglucuronidation rate, defined as the amount of formed aglycone per time per amount of total protein in the reaction, was determined with linear regression. Indomethacin-G and telmisartan-G were incubated in the presence of the bacterial lysates for a predetermined time, after which the formed aglycone was detected with ultraperformance liquid chromatography. For *Bacteroides uniformis* and *Bacteroides thetaiotaomicron*, the incubation times were 15 minutes, whereas *Phocaeicola vulgatus* was incubated with indomethacin-G for 5 minutes and telmisartan-G for 7.5 minutes. Here, the bars represent the mean of 3 technical replicates (shown as points) originating from a single experiment. Indomethacin-G, indomethacin acyl-*β*-d-glucuronide; 4-MU, 4-methylumbelliferone; 4-MUG, 4-methylumbelliferyl-*β*-d-glucuronide; telmisartan-G, telmisartan acyl-*β*-d-glucuronide.

To understand the distribution of GUS activity between commensal bacterial species, we measured 4-MUG deglucuronidation by 19 bacterial strains from 3 major phyla spanning 5 taxonomic orders (Bacteroidales, Bifidobacteriales, Eubacteriales, Lachnospirales, and Lactobacillales) (Fig. 2A). Only 4 isolates (*Bacteroides thetaiotaomicron*, *Bacteroides uniformis*, *Phocaeicola vulgatus*, and *Lachnospira* sp.) exhibited 4-MUG deglucuronidation activity. No deglucuronidation activity was detected for any the Bifidobacteriales, Eubacteriales, or Lactobacillales isolates. We then further compared the deglucuronidation activities of *B. thetaiotaomicron*, *B. uniformis*, and *P. vulgatus* using 4-MUG, indomethacin-G, telmisartan-G, and ezetimibe-G as GUS substrates. All 3 Bacteroidales isolates were capable of cleaving indomethacin-G and telmisartan-G (Supplemental Fig. 3), whereas no ezetimibe-G hydrolysis could be detected at up to 90 minutes of incubation (data not shown). Of the 3 tested Bacteroidales isolates, *P. vulgatus* showed the highest GUS activity toward the drug glucuronides and 4-MUG, whereas no striking difference was observed between the deglucuronidation activities of *B. uniformis* and *B. thetaiotaomicron* (Fig. 2B). Here, the greatest difference was detected in 4-MUG deglucuronidation, with the 4-MUG deglucuronidation rate by *P. vulgatus* being 8.9-fold the rate by *B. thetaiotaomicron*.

### 3.3 Association of individual characteristics with gut microbial deglucuronidation activity

Next, we sought out to examine relationships between donor characteristics (Supplemental Table 1) and fecal deglucuronidation activity. All donors reported a standard mixed diet consisting of meat, dairy, and vegetables; therefore, diet effects on deglucuronidation activity could not be examined. The observed deglucuronidation rates did not appear to correlate with the age or body mass index of the donors (Supplemental Table 5). Furthermore, there was no association between previous diagnoses or the use of dietary supplements or medicines and deglucuronidation activity (Supplemental Fig. 4). Only 2 donors reported the consumption of lactic acid bacteria (LAB) from a dietary supplement or LAB-fortified food, and thus the effects of LAB supplementation on deglucuronidation activity could not be examined.

**Fig. 3:**
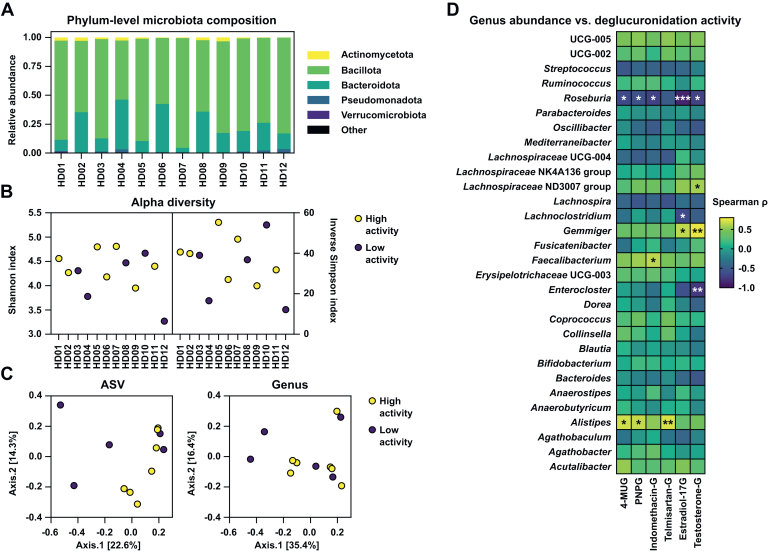
Phylum-level compositions (A) and the amplicon sequence variant (ASV)–level alpha diversities (B) of the fecal microbiotas of healthy donors HD01–HD12. Principal coordinate analysis of the microbiota compositions of HD01–HD12 at the ASV level and genus level (C) calculated with the Bray-Curtis dissimilarity index. Donors with high and low deglucuronidation activity are colored in yellow and purple, respectively, in the alpha and beta diversity plots. Spearman correlation of the relative abundance of the most abundant genera and deglucuronidation activity (D). In panel D, yellow corresponds to high *ρ* values and blue to low values. ∗*P* < .05, ∗∗*P* < .01, ∗∗∗*P* < .001. Presented are the unadjusted *P* values; see Supplemental Material 2 for adjusted (Benjamini-Hochberg) values. Estradiol-17G, *β*-estradiol 17-*β*-d-glucuronide; indomethacin-G, indomethacin acyl-*β*-d-glucuronide; 4-MUG, 4-methylumbelliferyl-*β*-d-glucuronide; PNPG, *p*-nitrophenyl-*β*-d-glucuronide; telmisartan-G, telmisartan acyl-*β*-d-glucuronide; testosterone-G, testosterone *β*-d-glucuronide.

We then characterized the donors’ fecal microbiotas with 16S rRNA gene sequencing to determine relationships between the fecal microbiota composition and deglucuronidation activity. The microbiotas of all donors were dominated by bacteria from the Bacillota and Bacteroidota phyla (Fig. 3A). There were no obvious differences in the alpha diversities of the individual samples or between high- and low-activity donors (Fig. 3B). However, there was a mostly nonsignificant trend for the diversity indices to positively correlate with deglucuronidation activity (Supplemental Fig. 5; Supplemental Table 6). Principal coordinate analysis of the relative abundances of amplicon sequence variants or taxa agglomerated at the genus level did not show clustering of the high- and low-activity donors’ microbiotas (Fig. 3C).

Instead, the relative abundances of several of the most abundant genera correlated with the deglucuronidation rates of the studied GUS substrates (Fig. 3D; Supplemental Material 2), including *Alistipes* with telmisartan-G (Spearman *ρ* = 0.713, 95% CI, 0.236–0.913, *P* = .00920), *Faecalibacterium* with indomethacin-G (Spearman *ρ* = 0.643, 95% CI, 0.110–0.889, *P* = .0240), and *Gemmiger* with testosterone-G (Spearman *ρ* = 0.750, 95% CI, 0.308–0.925, *P* = .00500); additional positive abundance-activity relationships were also identified at the species level (Supplemental Material 3). The abundance of *Roseburia* was negatively correlated with deglucuronidation activity, particularly for estradiol-17G (Spearman *ρ* = –0.864, 95% CI, –0.547 to −0.994, *P* = .000612). Furthermore, differential abundance analysis revealed 2-fold abundance of *Faecalibacterium* (*P* = .0303, Mann-Whitney *U* test) and 3-fold abundance of *Alistipes* (*P* = 0.106, Mann-Whitney *U* test) in the fecal microbiotas of high-activity donors compared with those of low-activity donors (Supplemental Material 4); at the species level, differences in the mean abundances of *Alistipes* spp. and *Faecalibacterium prausnitzii* between activity groups were also noted (Supplemental Material 5).

**Fig. 4:**
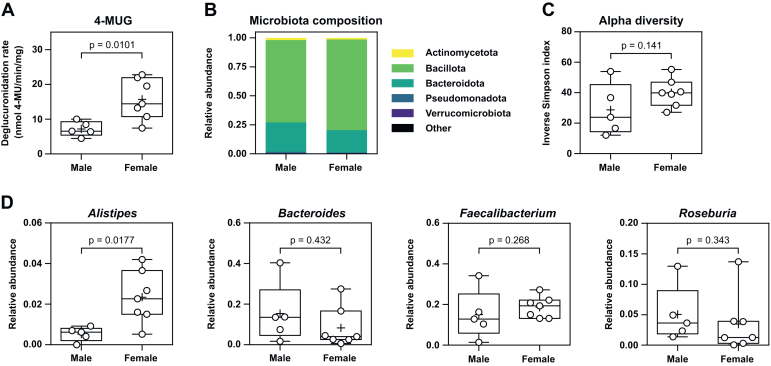
Deglucuronidation rate of 4-MUG grouped by sex of the healthy donors HD01–HD12 (A). Statistical significance of the difference in 4-MUG deglucuronidation rates between sexes was tested with the Mann-Whitney *U* test. Phylum-level microbiota compositions of HD01–HD12 averaged by sex (B). Alpha diversities of the microbiotas of donors HD01–HD12 grouped by sex (C). Statistical significance in the difference of alpha diversity indices between sexes was tested with the Kolmogorov-Smirnov test. Relative abundances of *Alistipes*, *Bacteroides*, *Faecalibacterium*, and *Roseburia* (D) in the fecal microbiotas of donors HD01–HD12 grouped by sex. The Mann-Whitney *U* test was used for the assessment of statistical significance in the difference in relative abundance between the sexes. Presented are the unadjusted *P* values; see Supplemental Material 6 for the adjusted (Benjamini-Hochberg) *P* values for the differential abundance analysis. The boxes extend from the 25th to the 75th percentile, with the line representing the median, the cross representing the mean, and the whiskers extending from the minimum to the maximum value. 4-MU, 4-methylumbelliferone; 4-MUG, 4-methylumbelliferyl-*β*-d-glucuronide.

Interestingly, samples from female donors exhibited approximately 2-fold average deglucuronidation rates of 4-MUG (*P* = .0101), PNPG (*P* = .0480), indomethacin-G (*P* = .0303), and telmisartan-G (*P* = .0480) when compared with samples collected from male donors (Mann-Whitney *U* test) (Fig. 4A; Supplemental Fig. 6; Supplemental Table 7). Although the average deglucuronidation rates of estradiol-17G and testosterone-G were also higher in females, there was high interindividual variability in the deglucuronidation rates of these steroid glucuronides in females and these differences did not thus reach statistical significance (Supplemental Fig. 6; Supplemental Table 7). There were no differences in phylum-level compositions or the diversity indices of the microbiotas of female and male donors (Fig. 4, B and C). However, the relative abundance of *Alistipes* was approximately 4.4-fold in female donors’ microbiotas compared with those of male donors (*P* = .0177, Mann-Whitney *U* test) (Fig. 4D and Supplemental Material 6). In contrast, the abundances of other known GUS-producing *Bacteroides*, *Faecalibacterium*, and *Roseburia* genera did not appear to be different between the sexes, although species-level analyses suggested higher abundance of *Roseburia inulinivorans* in males (*P* = .0480, Mann-Whitney *U* test) (Supplemental Material 7).

### 3.4 Intraindividual variability in gut microbial deglucuronidation activity

**Fig. 5:**
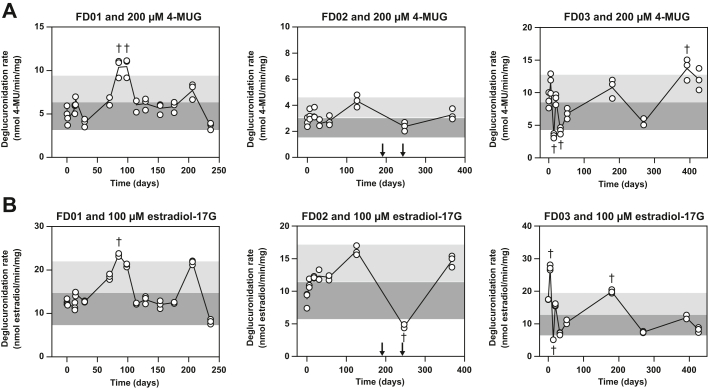
Deglucuronidation of 200 *μ*M 4-MUG (A) and 100 *μ*M estradiol-17G (B) in the presence of fecal lysates produced from the sequential fecal samples of 3 FMT donors FD01–FD03 (A). After the initiation of the reaction, 4-MU formation was followed at 1-minute intervals. The 4-MUG deglucuronidation rate, defined as the amount of formed aglycone per time per amount of total protein in the reaction, was determined with linear regression. The formed estradiol was measured with ultraperformance liquid chromatography after a 5-minute incubation. The 4-MUG data were collected from 3 individual experiments shown as individual data points. The estradiol data are from a single experiment conducted in triplicate samples shown as individual data points. For both substrates, the line connects the mean deglucuronidation rates of the different sample collection time points. The highlighted area represents the mean ± 50% of the deglucuronidation rates of the different fecal sample collection time points. The dagger symbol (†) denotes fecal sample collection time points where the deglucuronidation rate deviates from the mean deglucuronidation rate of all time points by >50%. Time points where donor FD02 underwent oral antibiotic treatment (amoxicillin 500 mg 3 times daily on days 188–194 and cefalexin 500 mg twice daily on days 239–246, after which they quit as a FMT donor) are shown as arrows. estradiol-17G, *β*-estradiol 17-*β*-d-glucuronide; FMT, fecal microbiota transplantation; 4-MU, 4-methylumbelliferone; 4-MUG, 4-methylumbelliferyl-*β*-d-glucuronide.

Intraindividual variability in gut microbial deglucuronidation activity was evaluated with fecal lysates produced from sequential fecal samples collected from 3 FMT donors. First, using 4-MUG at 200 *μ*M as a GUS substrate, the fecal deglucuronidation activities of the 3 FMT donors appeared to be relatively stable over time (Fig. 5A). FD03 exhibited the highest intraindividual variability of the 3 donors (Table 2); however, the observation period for this donor was also the longest. We then evaluated the intraindividual variability in estradiol-17G deglucuronidation (Fig. 5B) because this GUS substrate exhibited high interindividual variability compared with 4-MUG. Although the variability metrics were essentially unchanged for donor FD01, they were slightly increased for donors FD02 and FD03 (Table 2). Notably, FD02 showed a decrease in estradiol-17G deglucuronidation activity after antibiotic treatment (Fig. 5B). However, interindividual variability in estradiol-17G deglucuronidation still notably exceeded its intraindividual variability.

**Table 2:** Measures of intraindividual variability in the processing of 200 *μ*M 4-MUG and 100 *μ*M estradiol-17G by fecal lysates produced from sequential fecal samples collected from 3 healthy fecal microbiota transplantation donors (FD01–FD03) The individual samples’ deglucuronidation activities are presented in Fig. 5.

	FD01 (*n* = 14)	FD02 (*n* = 8)	FD03 (*n* = 10)
**200*****μ*****M4-MUG**			
Mean (SD) (nmol/min per mg)	6.34 (2.04)	3.08 (0.622)	8.57 (3.53)
CV (%)	32.1	20.2	41.2
Range (nmol/min per mg)	3.62–10.5	2.37–4.33	3.40–13.7
Maximum/minimum fold	2.89	1.83	4.03
**100*****μ*****M estradiol-17G**			
Mean (SD) (nmol/min per mg)	14.7 (4.57)	11.5 (3.57)	13.0 (6.97)
CV (%)	31.0	31.1	53.4
Range (nmol/min per mg)	8.12–23.5	4.57–16.2	5.12–27.2
Maximum/minimum fold	2.89	3.54	5.32

estradiol-17G, *β*-estradiol 17-*β*-d-glucuronide; 4-MUG, 4-methylumbelliferyl-*β*-d-glucuronide.

### 3.5 Effect of FMT on gut microbial deglucuronidation activity

**Fig. 6:**
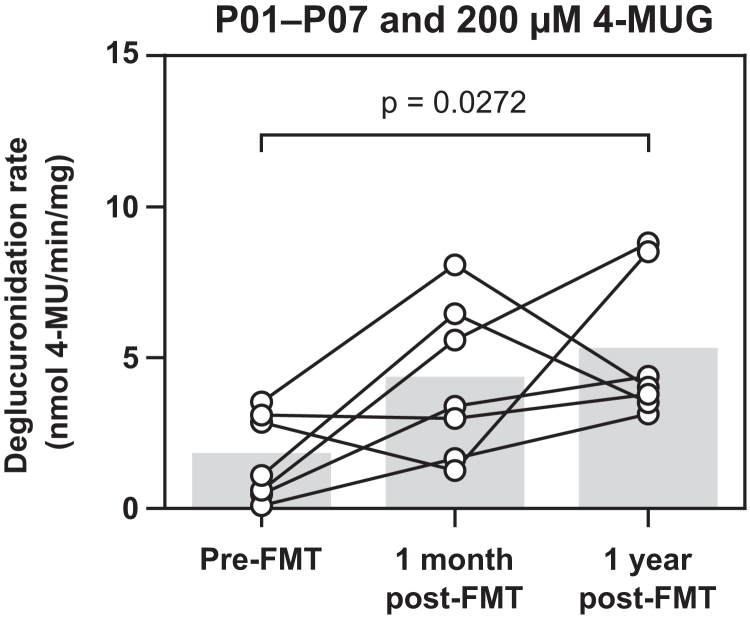
Deglucuronidation of 200 *μ*M 4-MUG by fecal lysates produced from the fecal samples of patients P01–P07 with recurrent *Clostridioides difficile* infection before FMT, 1 month after FMT, and 1 year after FMT. After the initiation of the reaction, 4-MU formation was followed at 1-minute intervals. The 4-MUG deglucuronidation rate, defined as the amount of formed aglycone per time per amount of total protein in the reaction, was determined with linear regression. The data were collected from 3 independent experiments, the means of which for each patient are shown as individual data points. The bars represent the mean deglucuronidation rates of all lysates at the different fecal sample collection time points. The lines connect the deglucuronidation rates of the individual patients. Statistical significance of the difference between the means was tested with the Friedman test with Dunn post hoc test for multiple comparisons. FMT, fecal microbiota transplantation; 4-MU, 4-methylumbelliferone; 4-MUG, 4-methylumbelliferyl-*β*-d-glucuronide.

To evaluate the effect of FMT on gut microbial deglucuronidation activity, we studied the deglucuronidation of 4-MUG with lysates prepared from fecal samples collected from patients with rCDI (*n =* 7) before FMT, 1 month after FMT, and 1 year after FMT (Fig. 6). In this cohort, FMT was associated with an increase in fecal deglucuronidation activity (*P* = .0272, Friedman test with Dunn post hoc test): the mean 4-MUG deglucuronidation rate of all patients 1 year after FMT was 3-fold the rate measured before FMT. The 4 patients with the lowest pre-FMT deglucuronidation activities exhibited the clearest increases in the 4-MUG deglucuronidation rate, with the 1-year post-FMT activity of P02 being 29-fold compared with the pre-FMT activity (Supplemental Table 8). In contrast, 2 patients with relatively high pre-FMT deglucuronidation activity exhibited essentially unchanged pre-FMT and 1-year post-FMT 4-MUG deglucuronidation rates. In addition, the increase in deglucuronidation activity was associated with a decrease in the relative interindividual variability (Table 3).

**Table 3:** Measures of interindividual variability in the processing of 200 *μ*M 4-methylumbelliferyl-*β*-d-glucuronide in the presence of fecal lysates produced from fecal samples of recurrent *Clostridioides difficile* infection patients (*n* = 7) before FMT, 1 month after FMT, and 1 year after FMT The individual samples’ deglucuronidation activities are presented in Fig. 6.

	Pre-FMT	1 month after FMT	1 year after FMT
Mean (SD) (nmol/min per mg)	1.68 (1.43)	4.20 (2.56)	5.16 (2.42)
CV (%)	84.8	60.9	46.9
Range (nmol/min per mg)	0.107–3.53	1.23–8.07	3.13–8.81
Maximum/minimum fold	33.1	6.39	2.82

FMT, fecal microbiota transplantation.

## 4 Discussion

Glucuronidation contributes to the formation of major drug metabolites for 17% of the most prescribed oral drugs,^44^ which if excreted back into the intestine as glucuronide conjugates may be cleaved by GUS enzymes. Previous studies have linked deglucuronidation activity to exposure and/or intestinal toxicity of, for example, the irinotecan metabolite SN-38^16^^,^^45^^,^^46^ and mycophenolic acid (MPA),^4^^,^^5^^,^^47^, ^48^, ^49^ highlighting that variability in deglucuronidation activity may affect drug therapy. Similarly, GUS-mediated cleavage of nonsteroidal anti-inflammatory drug (NSAID) acyl glucuronides, such as indomethacin-G, may contribute to NSAID-associated enteropathy.^50^, ^51^, ^52^ In addition, gut microbial deglucuronidation activity and EHR may play a role in the individual variability in exposure and effects of several other drugs such as ezetimibe^53^, ^54^, ^55^ and telmisartan.^56^^,^^57^

Consistent with previous literature,^8^^,^^10^^,^^11^^,^^17^^,^^18^ our results demonstrate that GUS enzymes expressed by the fecal microbiota reactivate glucuronidated drugs and endobiotics, although the fecal deglucuronidation activity varies between individuals. To better understand how this variability links to drug response and pharmacokinetic parameters, future clinical studies on drugs that undergo EHR could include fecal sampling and deglucuronidation activity measurement in tandem with pharmacokinetic and/or pharmacodynamic evaluation. Previously, high fecal deglucuronidation activity has been associated with prolonged course of diarrhea in kidney transplantation patients treated with mycophenolate mofetil.^49^ However, although GUS inhibition has been shown to protect against NSAID-induced enteropathy in mice,^52^ it remains to be determined whether fecal deglucuronidation activity would predict the severity of intestinal ulceration induced by indomethacin in humans.

Although our small sample size did not allow us to determine relationships between most donor characteristics and deglucuronidation activity, we were able to identify some associations between fecal microbial communities and deglucuronidation activity. The observed trend for higher deglucuronidation activity in samples with higher alpha diversity is supported by a previous report on a larger dataset.^58^ Furthermore, corroborating the work by Flores et al,^58^ the relative abundance of *Alistipes* was positively correlated with GUS activity. Although not characterized in detail, *Alistipes* GUS enzymes have been identified from human fecal microbiomes,^9^ and the presence of *Alistipes putredinis* predicts low gut proteolytic activity via the production of unconjugated bilirubin.^59^ Altogether, these data suggest a possible important role for *Alistipes* spp. in gut microbial deglucuronidation.

In contrast, while *Roseburia* GUS enzymes have been identified and characterized,^8^^,^^10^^,^^14^^,^^60^ we observed a negative correlation between *Roseburia* abundance and deglucuronidation activity, similar to Flores et al.^58^ Moreover, our *Roseburia* sp. isolate did not exhibit deglucuronidation activity similar to the *R. inulinivorans* DSM 16841 type strain studied previously,^61^ although *R. inulinivorans* GUS has been shown to cleave 4-MUG, MPA *β*-d-glucuronide, and hormone glucuronides.^8^^,^^10^ These data thus suggest considerable strain variability in deglucuronidation activity. Although *Bacteroides* abundance did not positively correlate with fecal deglucuronidation activity, *Bacteroides* GUS enzymes of varying structures have been characterized previously.^8^^,^^10^^,^^12^^,^^13^^,^^15^^,^^16^ However, we observed notable differences in the deglucuronidation activities between Bacteroidales species, and among the 7 tested strains, only 3 (*B. thetaiotaomicron*, *B. uniformis*, and *P. vulgatus*) showed deglucuronidation activity. In line with this, it was recently reported that bacterial communities dominated by *B. uniformis* and *P. vulgatus* link to enhanced exposure of MPA, highlighting the clinical significance of these bacteria in its EHR.^4^^,^^5^

We observed higher deglucuronidation activity in samples from female donors compared with male donors. A previous study on 1793 gut metagenomes reported a slight difference in the mean number of GUS genes between males and females with males showing higher levels, although GUS read abundances were highly variable in both sexes.^62^ Here, of the GUS-associated taxa that we observed to be linked with fecal deglucuronidation activity, *Alistipes* abundance appeared different between sexes. These data suggest that differences in the gut microbiome may contribute to the sex differences in the plasma levels of glucuronidated compounds, such as telmisartan.^63^ Although Hirvensalo et al^63^ hypothesized that higher glucuronidation capacity in men could cause the higher telmisartan-G/telmisartan area under the curve ratio in men, higher deglucuronidation activity in female subjects could provide an additional explanation for the higher telmisartan *C*_max_ and area under the curve observed in females.

The interindividual variability in fecal deglucuronidation activity appears to be substrate dependent, which may be explained by the selectivity of GUS enzymes based on the size, shape, and polarity of the aglycone.^13^ The pan-GUS reporter substrates with small and polar aglycones showed less variability in deglucuronidation compared with steroid glucuronides, which contain larger nonpolar aglycone structures and are preferentially cleaved by select GUS isoforms.^8^ Thus, the observed high variability in steroid glucuronide processing, also described previously,^8^ could result from interindividual differences in the abundance of these specific enzymes. However, variability in deglucuronidation was similar between the acyl glucuronides and GUS reporter substrates, suggesting that these ester-linked glucuronides are also efficiently cleaved by various GUS isoforms despite their larger aglycone structure. Altogether, these data suggest that pan-GUS reporter substrates may underestimate the variability in the deglucuronidation of specific substrates that are processed only by select enzyme isoforms.

The cleavage of indomethacin-G and telmisartan-G by the fecal and bacterial isolate lysates was expected, because both are excreted in feces mainly in an unconjugated form.^64^^,^^65^ However, despite ezetimibe-G also seemingly being deglucuronidated in the gut,^54^ we were unable to detect ezetimibe formation in any of our in vitro studies, although glucuronidase treatment has been used for the analysis of total ezetimibe from clinical samples.^54^^,^^66^ Furthermore, ezetimibe-G only modestly inhibited 4-MUG deglucuronidation. Although it may be that ezetimibe-G is only hydrolyzed by a small subset of GUS enzymes present at low levels in feces, the racemic nature of the used ezetimibe-G stock may have also interfered with the experimental setup. However, a previous study similarly observed no regorafenib-G cleavage in human or murine feces despite intestinal homogenates of specific pathogen-free but not germ-free mice showing regorafenib-G deglucuronidation activity.^14^ This further suggests that some glucuronides may be poorly hydrolyzed in the fecal deglucuronidation assay, possibly due to low abundance of specific GUS enzymes in feces.

Our finding that fecal deglucuronidation activity is relatively stable within individuals and less variable than between individuals is supported by the longitudinal stability of the fecal microbial composition^19^^,^^30^ and a previous considerably shorter study following fecal deglucuronidation activity in elderly subjects.^23^ Importantly, the high interindividual variability observed in estradiol-17G deglucuronidation was not reflected in intraindividual variability, suggesting an important homeostatic role for the taxa responsible for endobiotic glucuronide processing. Although the intraindividual variability in drug glucuronide processing by the gut microbiota can still contribute to the intraindividual pharmacokinetic and pharmacodynamic variability of drugs undergoing EHR, other physiological processes involved in EHR, such as gall bladder emptying, may play a bigger role in day-to-day variation.

Previously, intestinal diseases have been shown to be differentially associated with fecal deglucuronidation activity. Although this activity appears to be lower in patients with Crohn's disease and irritable bowel syndrome compared with controls,^59^^,^^67^^,^^68^ colon cancer has been associated with increased activity.^69^ Here, we observed low fecal deglucuronidation activities in patients with rCDI before FMT, which likely results from the multiple courses of antibiotics administered to these patients before FMT leading to significantly altered microbiota composition and reduced diversity compared with healthy subjects.^30^ After FMT, however, the gut microbiotas of patients with rCDI resembled those of the healthy donors,^30^ which was reflected in the improvement of deglucuronidation activity. Resistance of a heathy gut microbiome to changes caused by antibiotics^30^ may explain why oral antibiotic treatment only slightly decreased 4-MUG deglucuronidation activity in donor FD02. In addition, while the decrease in estradiol-G deglucuronidation rate in FD02 after antibiotic treatment was more pronounced compared with 4-MUG, the deglucuronidation activity was restored by the next sample collection time point.

The main limitation of our study is its limited sample size, which likely does not capture the full extent of individual variability in fecal deglucuronidation activity. Furthermore, although the 16S rRNA gene amplicon sequencing gives us information about the overall fecal microbiota composition, it does not produce functional gene information and lacks the depth for identifying all possible associations between taxa abundance and deglucuronidation activity. The identified associations between individual characteristics and activity should thus be considered exploratory findings that warrant confirmation by future studies on larger populations using more comprehensive microbiome characterization methods. However, even species-level microbiota composition may not correlate with fecal deglucuronidation activity because GUS expression can vary at the strain level within the same species, apparent for *R. inulinivorans* as discussed above. Determination of GUS protein abundance over bacterial composition or GUS gene abundance may thus be required to accurately predict deglucuronidation activity.^10^ Moreover, because some glucuronides are preferentially cleaved by certain structural GUS isoforms, statistical analyses may need to be very restricted to detect significant correlations between GUS protein abundance and fecal deglucuronidation activity.^31^

Finally, the microbial composition of feces does not reflect the composition of the small intestine, which may favor select fast-growing bile-tolerant facultative anaerobes.^70^ However, because of the low volume of small intestinal contents, we were unable to collect sufficient samples for small intestinal deglucuronidation activity studies. Because the small intestine is the major site for drug absorption as well as the site for bile excretion, regarding EHR, it would be important to characterize the deglucuronidation activity of small intestinal bacterial communities in addition to those present in feces.

## 5 Conclusion

Although deglucuronidation is a function of a healthy gut microbiota, it may be subject to individual variability, which could have pharmacokinetic and pharmacodynamic consequences for glucuronidated drugs undergoing EHR. The extent of interindividual variability in fecal deglucuronidation activity appears to be substrate dependent, and specific bacterial taxa likely contribute to the high activity observed in select samples. Major compositional changes in the gut microbiota composition by, for example, repeated antibiotic courses or FMT, can substantially modulate the fecal deglucuronidation activity, which otherwise appears to be relatively stable within healthy individuals. Future studies should investigate the role of small intestinal bacterial communities in gut microbial deglucuronidation and EHR.

## Conflict of interest

Oona Neulasalmi reports a relationship with Eli Lilly and Company that includes employment. The employer had no role in designing, implementing, or reporting of this study. All other authors declare no conflicts of interest.
