# Supplementary material for: Characterization of fecal deglucuronidation activity in healthy subjects and in patients treated with fecal microbiota transplantation
Source: Drug Metab Dispos. 2025 Nov 20;53(12):100205. doi: 10.1016/j.dmd.2025.100205 (PMC12799528; doi:10.1016/j.dmd.2025.100205)
Supplement: Supplementary Material — Appendix A [file mmc1.docx]

**SUPPLEMENTAL MATERIALS**

**Characterization of fecal deglucuronidation activity in healthy subjects and in patients treated with fecal microbiota transplantation**

Arttu Uoti, Oona Neulasalmi, Kaisa Hiippala, Timo Oksanen, Perttu Arkkila, Lauri Puustinen, Reetta Satokari, Noora Sjöstedt *

* Corresponding author ([noora.sjostedt@helsinki.fi](mailto:noora.sjostedt@helsinki.fi))

Table of contents

[**Supplemental Material 1.** Microbiota analysis. 2](#_Toc216036821)

[**Supplemental Table 1**. Characteristics of the fecal sample donors HD01–HD12. 6](#_Toc216036822)

[**Supplemental Table 2.** Growth conditions of the bacterial isolates investigated in this study. 7](#_Toc216036823)

[**Supplemental Table 3.** Number of forward reads remaining after each preprocessing step. 8](#_Toc216036824)

[**Supplemental Figure 1.** Deglucuronidation of drug and steroid glucuronides by pooled fecal lysate. 9](#_Toc216036825)

[**Supplemental Figure 2.** Inhibition of 4-MUG deglucuronidation by ezetimibe-G. 10](#_Toc216036826)

[**Supplemental Table 4.** Correlation of deglucuronidation rates. 11](#_Toc216036827)

[**Supplemental Figure 3.** Deglucuronidation of drug glucuronides by *Bacteroidales* isolates. 12](#_Toc216036828)

[**Supplemental Table 5.** Correlation of deglucuronidation rates with age and BMI. 13](#_Toc216036829)

[**Supplemental Figure 4.** Association of medical diagnoses and medication and supplement use with deglucuronidation activity. 14](#_Toc216036830)

[**Supplemental Figure 5.** Association of alpha diversity indices with deglucuronidation activity. 15](#_Toc216036831)

[**Supplemental Table 6.** Correlation of deglucuronidation rates with alpha diversity indices. 16](#_Toc216036832)

[**Supplemental Figure 6.** Sex-dependent deglucuronidation activity. 17](#_Toc216036833)

[**Supplemental Table 7.** Sex-dependent deglucuronidation activity. 18](#_Toc216036834)

[**Supplemental Table 8.** Effect of fecal microbiota transplantation on 4-MUG deglucuronidation. 19](#_Toc216036835)

# **Supplemental Material 1.** Microbiota analysis.

PCR primers were trimmed from the raw forward (FWD) and reverse (REV) reads using Cutadapt. Untrimmed reads were discarded.

python -m cutadapt -g CCTACGGGNGGCWGCAG -G GACTACHVGGGTATCTAATCC --discard-untrimmed -o output.FWD.fastq.gz -p output.REV.fastq.gz input.FWD.fastq.gz input.REV.fastq.gz

DADA2 was used for further filtering, trimming, and denoising of the forward reads using standard parameters.

out <- filterAndTrim(fnFs, filtFs, truncLen = 280, maxN = 0, maxEE = 2, truncQ = 2, rm.phix = TRUE, compress = TRUE, multithread = FALSE)

errF <- learnErrors(filtFs, multithread = TRUE)

filtFs <- filtFs[file.exists(filtFs)]

dadaFs <- dada(filtFs, err = errF, multithread = TRUE)

Chimeric sequences were removed from the sequence table.

seqtab <- makeSequenceTable(dadaFs)

seqtab.nochim <- removeBimeraDenovo(seqtab, method = "consensus", multithread = TRUE, verbose = TRUE)

Taxonomic assignments up to the Genus level were achieved with a naïve Bayesian classifier algorithm included in DADA2 and Silva training data. Further Species-level assignments were based on exact matching.

taxa <- assignTaxonomy(seqtab.nochim, silvaGenusPath, multithread = TRUE)

taxa <- addSpecies(taxa, silvaSpeciesPath)

The number of reads per each fecal DNA sample remaining after each preprocessing step with Cutadapt and DADA2 are presented in Supplemental Table 3.

The DADA2 outputs were then used to construct a phyloseq object.

ps <- phyloseq(otu_table(seqtab.nochim, taxa_are_rows = FALSE), sample_data(samdf), tax_table(taxa))

Alpha diversity indices were calculated from the phyloseq object without prior filtering of low-abundance taxa using microbiome.

diversities <- microbiome::alpha(ps, index = c("diversity_shannon", "diversity_inverse_simpson"))

The alpha diversity indices were correlated with deglucuronidation rates (stored in “processRates”) with Benjamini-Hochberg (BH) correction for p-values using psych.

diversity_correlation <- corr.test(x = processRates, y = diversities, method = "spearman", adjust = "BH", alpha = 0.05)

Low-abundance ASVs and ASVs without a Phylum-level classification were then filtered using phyloseq.

ps.f <- subset_taxa(ps, !is.na(Phylum))

ps.f <- filter_taxa(ps.f, function(x) sum(x) / sum(taxa_sums(ps.f)) >= 0.0001, prune = TRUE)

The filtered ASV counts were transformed into relative abundances using microbiome.

ps.relative <- microbiome::transform(ps.f, "compositional")

Rare phyla were agglomerated into “Other” using microbiome for visualization.

ps.relative.phyla <- aggregate_rare(ps.relative, level = "Phylum", detection = 0.5/100, prevalence = 1/12, include.lowest = TRUE)

Principal coordinate analysis (PCoA) was conducted on ASV relative abundances with Bray-Curtis distance using phyloseq.

ordination_bc <- ordinate(ps.relative, method = "PCoA", distance = "bray")

The taxa were then agglomerated at the genus level with taxa without a genus-level classification agglomerated as “Unknown” using microbiome.

ps.relative.genus <- aggregate_taxa(ps.relative, level = "Genus")

The genus-level relative abundances were then used for a PCoA with Bray-Curtis distance using phyloseq.

ordination_bc_genus <- ordinate(ps.relative.genus, method = "PCoA", distance = "bray")

The most abundant genera (0.05% detection, 83% prevalence) were selected for further analyses using microbiome.

ps.relative.genus.2 <- aggregate_rare(ps.relative, level = "Genus", detection = 0.05/100, prevalence = 10/12, include.lowest = TRUE)

Genera classified as “Unknown” or “Other” were excluded using phyloseq.

ps.relative.genus.2 <- prune_taxa(taxa_names(ps.relative.genus.2) != "Unknown", ps.relative.genus.2)

ps.relative.genus.2 <- prune_taxa(taxa_names(ps.relative.genus.2) != "Other", ps.relative.genus.2)

The abundance matrix was retrieved using microbiome.

ps.relative.genus.2.abundances <- as.data.frame(t(abundances(ps.relative.genus.2)))

The relative abundances of the most abundant genera were correlated with deglucuronidation rates with Benjamini-Hochberg (BH) correction for p-values using psych.

genus_correlation <- corr.test(x = processRates, y = ps.relative.genus.2.abundances, method = "spearman", adjust = "BH", alpha = 0.05)

Next, a new phyloseq object with the full species name for identified species was generated.

ps.relative.species <- ps.relative

tax <- as.data.frame(tax_table(ps.relative.species))

tax$Species <- ifelse(!is.na(tax$Species), paste(tax$Genus, tax$Species), tax$Species)

tax_table(ps.relative.species) <- as.matrix(tax)

The most abundant species (0.01% detection, 50% prevalence) were selected for further analyses using microbiome:

ps.relative.species <- aggregate_rare(ps.relative.species, level = "Species", detection = 0.01/100, prevalence = 6/12, include.lowest = TRUE)

Species classified as “Unknown” or “Other” were excluded using phyloseq.

ps.relative.species <- prune_taxa(taxa_names(ps.relative.species) != "Unknown", ps.relative.species)

ps.relative.species <- prune_taxa(taxa_names(ps.relative.species) != "Other", ps.relative.species)

The abundance matrix was retrieved using microbiome.

ps.relative.species.abundances <- as.data.frame(t(abundances(ps.relative.species)))

The relative abundances of the most abundant species were correlated with deglucuronidation rates with Benjamini-Hochberg (BH) correction for p-values using psych.

species_correlation <- corr.test(x = processRates, y = ps.relative.species.abundances, method = "spearman", adjust = "BH", alpha = 0.05)

Finally, a differential abundance analysis of genus and species relative abundances between high- and low-activity donors and between sexes (both here as “Group”) was conducted with the Mann-Whitney U test (Wilcoxon test). Here, the species-level analysis is showed as an example.

abund_df <- ps.relative.species.abundances

abund_df$Group <- sample_data(ps.relative.species)$Group

pvals.Group <- sapply(abund_df[, !(names(abund_df) %in% "Group")], function(x) {wilcox.test(x ~ abund_df$Group)$p.value})

adj.pvals.Group <- p.adjust(pvals.Group, method = "BH")

results.Group <- data.frame(Taxon = names(pvals.Group), P_Value = pvals.Group, Adjusted_P = adj.pvals.Group)

**Supplemental Table 1**. Characteristics of the fecal sample donors HD01–HD12. Age and body mass index are described as the median (range), whereas other characteristics are reported as the number of study subjects (% of all).

| **Age** | Year, median (range) | 36 (26–63) |
| --- | --- | --- |
| **Sex** | Female | 7 (58.3%) |
|  | Male | 5 (41.7%) |
| **Body mass index** | kg/m^2^, median (range) | 23.9 (20.4–28.3) |
| **Tobacco smoking** | Daily | 1 (8.33%) |
|  | None | 11 (91.7%) |
| **Alcohol consumption** | Daily | 1 (8.33%) |
|  | Weekly | 2 (16.7%) |
|  | Monthly or less | 8 (66.7%) |
|  | None | 1 (8.33%) |
| **Dietary groups consumed within the past two weeks** | Meat | 12 (100%) |
|  | Fish | 10 (83.3%) |
|  | Dairy | 12 (100%) |
|  | Eggs | 9 (75%) |
|  | Grains | 11 (91.7%) |
|  | Beans and legumes | 11 (91.7%) |
|  | Vegetables, fruits, and berries | 12 (100%) |
|  | Sweets and sodas | 10 (83.3%) |
| **Dietary supplements used within the past two weeks** | Any dietary supplement | 9 (75%) |
|  | Vitamin D | 4 (33.3%) |
|  | Omega-3 | 3 (25%) |
|  | Lactic acid bacteria ^a^ | 2 (16.7%) |
|  | Vitamin C | 2 (16.7%) |
|  | Biotin | 1 (8.33%) |
|  | Collagen | 1 (8.33%) |
|  | Multivitamin | 1 (8.33%) |
|  | Vitamin B | 1 (8.33%) |
|  | Zinc | 1 (8.33%) |
| **Diagnoses** | Any diagnosis | 6 (50%) |
|  | Multiple diagnoses | 4 (33.3%) |
|  | Irritable bowel syndrome ^b^ | 2 (16.7%) |
|  | Lactose intolerance | 2 (16.7%) |
|  | Hypercholesterolemia | 2 (16.7%) |
|  | Hypothyroidism | 2 (16.7%) |
|  | Other diagnosis | 2 (16.7%) |
| **Medicines used within the past two weeks** | Any medicine | 9 (75%) |
|  | Multiple medicines | 6 (50%) |
|  | Ibuprofen | 7 (58.3%) |
|  | Paracetamol | 3 (25%) |
|  | Levothyroxine | 2 (16.7%) |
|  | Other medicine(s) | 5 (41.7%) |

^a^ = supplements and fortified foods counted.

^b^ = diagnosed and self-diagnosed cases counted; donors asymptomatic at the time of fecal sample donation.

**Supplemental Table 2.** Growth conditions of the bacterial isolates investigated in this study. GAM = Gifu Anaerobic Medium, FAA = Fastidious Anaerobe Agar, FAB = Fastidious Anaerobe Broth, MRS = De Man-Rogosa-Sharpe, YBHI = Yeast Extract Brain-Heart Infusion, CO = Columbia Agar, BHI = Brain-Heart Infusion.

| **Isolate** | **Agar** | **Broth** | **Growth time** | **Anaerobic requirements** |
| --- | --- | --- | --- | --- |
| *Bacteroides caccae* | GAM | GAM | 48 h | not strict |
| *Bacteroides intestinalis* | GAM | GAM | 48 h | not strict |
| *Bacteroides thetaiotaomicron* | FAA | FAB | 48 h | not strict |
| *Bacteroides uniformis* | GAM | GAM | 48 h | not strict |
| *Phocaeicola vulgatus* | GAM | GAM | 48 h | not strict |
| *Odoribacter splanchnicus* | GAM | GAM | 48 h | not strict |
| *Parabacteroides distasonis* | GAM | GAM | 48 h | not strict |
| *Bifidobacterium adolescentis* | MRS | MRS | 48 h | not strict |
| *Bifidobacterium catenulatum* | MRS | MRS | 48 h | not strict |
| *Bifidobacterium longum 1* | MRS | MRS | 48 h | not strict |
| *Bifidobacterium longum 2* | MRS | MRS | 48 h | not strict |
| *Anaerotruncus* sp. | YBHI | YBHI | 48–72 h | strict |
| *Pseudoruminococcus massiliensis* | FAA, YBHI | YBHI | 48–72 h | strict |
| *Ruminococcoides* sp. | YBHI | YBHI | 48–72 h | strict |
| *Blautia* sp. | YBHI | YBHI | 48–72 h | strict |
| *Lachnospira* sp. | YBHI | YBHI | 48–72 h | strict |
| *Roseburia* sp. | FAA, YBHI | YBHI | 48–72 h | strict |
| *Enterococcus faecalis* | CO | BHI | 48 h | aerobic |
| *Enterococcus faecium* | CO | BHI | 48 h | aerobic |

# **Supplemental Table 3.** Number of forward reads remaining after each preprocessing step.

| **Sample ID** | **Raw reads** | **After**  **Cutadapt** | **After**  **filtering** | **After**  **denoising** | **After**  **chimera removal** | **% remaining** |
| --- | --- | --- | --- | --- | --- | --- |
| HD01 | 274306 | 270014 | 240692 | 231682 | 174596 | 63.7 |
| HD02 | 280812 | 277340 | 247529 | 242616 | 180324 | 64.2 |
| HD03 | 261565 | 258407 | 231564 | 225637 | 161612 | 61.8 |
| HD04 | 261295 | 258083 | 228738 | 224929 | 170987 | 65.4 |
| HD05 | 293573 | 289820 | 258668 | 249171 | 184543 | 62.9 |
| HD06 | 357303 | 352394 | 315197 | 308186 | 220481 | 61.7 |
| HD07 | 177992 | 175872 | 158144 | 157268 | 151085 | 84.9 |
| HD08 | 277500 | 271126 | 241534 | 234351 | 170468 | 61.4 |
| HD09 | 261064 | 257783 | 230478 | 226176 | 165133 | 63.3 |
| HD10 | 287906 | 284353 | 254662 | 245869 | 172872 | 60.0 |
| HD11 | 278396 | 275027 | 244702 | 238037 | 170811 | 61.4 |
| HD12 | 268425 | 265481 | 235158 | 232640 | 167403 | 62.4 |


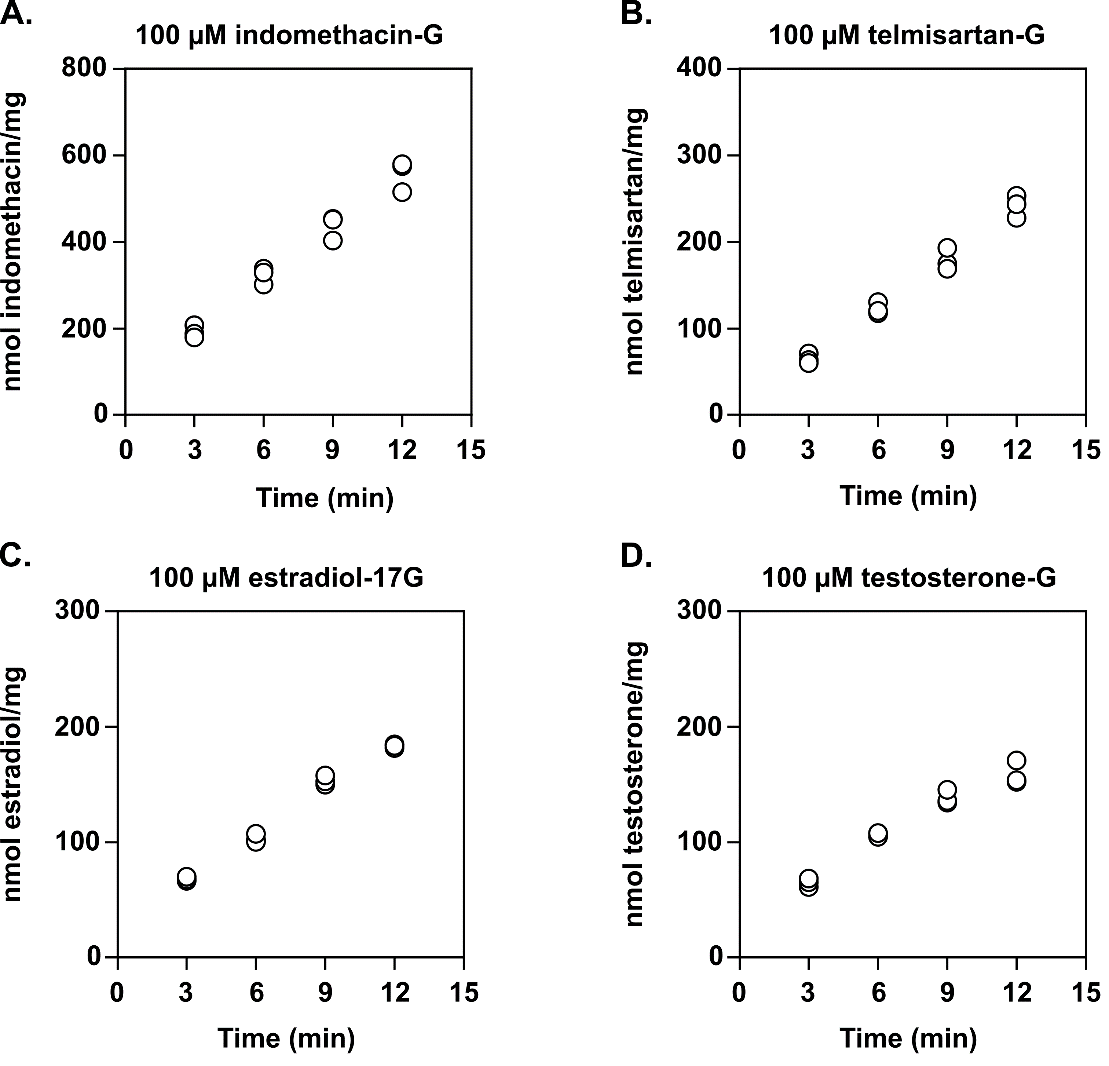


**Supplemental Figure 1.** Deglucuronidation of drug and steroid glucuronides by pooled fecal lysate. Indomethacin-G (A), telmisartan-G (B), estradiol-17G (C), and testosterone-G (D) were incubated at 100 µM with a pooled fecal lysate produced from the fecal samples of donors HD01–HD05. The reactions were stopped at 3, 6, 9, and 9 min by the addition of ice-cold acetonitrile, after which the formed aglycones were measured with UPLC. Presented are the individual triplicate values originating from a single experiment normalized to the amount of total protein (5 µg) of the reaction.


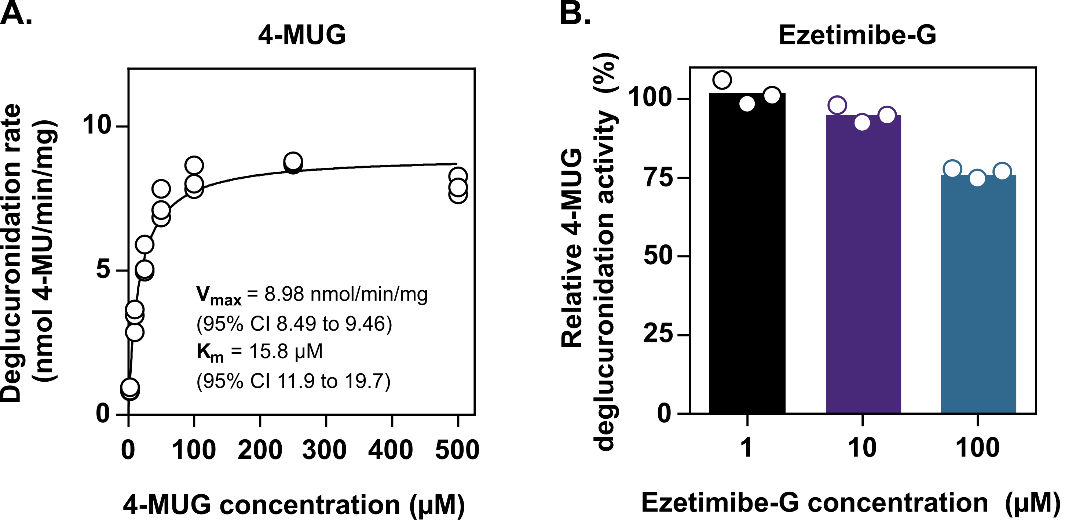


**Supplemental Figure 2.** Inhibition of 4-MUG deglucuronidation by ezetimibe-G. First, kinetic parameters for 4-MUG deglucuronidation by pooled fecal lysate were determined (A). Pooled fecal lysate (10 µg total protein per reaction) from donors HD01–HD05 was incubated with different concentrations of 4-MUG at +37 °C and the 4-MUG deglucuronidation rates were then calculated as described in Materials and Methods. Michaelis-Menten parameters V_max_ and K_m_ were calculated using GraphPad Prism with the least squares fitting method. The data were collected from a single experiment with triplicate samples shown as individual points with the line representing the fitting of the data to the Michaelis-Menten equation. Then, the inhibitory potential of ezetimibe-G on 4-MUG deglucuronidation was evaluated (B). Pooled fecal lysate (5 µg total protein per reaction) from donors HD01–HD05 was incubated with 10 µM 4-MUG and 1, 10, and 100 µM ezetimibe-G or DMSO (vehicle control) at +37 °C. The 4-MUG deglucuronidation rates calculated as described in Materials and Methods at the different ezetimibe-G concentrations were then normalized to the rate measured in the presence of vehicle only. The data were collected from a single experiment performed in triplicate samples shown as individual data points with the bar representing the mean.

**Supplemental Table 4.** Correlation of deglucuronidation rates. Spearman correlation was used to correlate the deglucuronidation rates of 200 µM 4-MUG, 500 µM PNPG, and 100 µM indomethacin-G, telmisartan-G, estradiol-17G, and testosterone-G. CI = 95% confidence interval, ρ = Spearman correlation coefficient, sig = significance, ns = not significant, * = p < 0.05, ** = p < 0.01, *** = p < 0.001, p.adj = Benjamini-Hochberg-adjusted p-value for multiple comparisons.

| **Correlation** | **lower CI** | **ρ** | **upper CI** | **p** | **sig** | **p.adj** |
| --- | --- | --- | --- | --- | --- | --- |
| 4-MUG – PNPG | 0.565 | 0.860 | 0.960 | 3.32  E-04 | *** | 9.95  E-04 |
| 4-MUG – Indomethacin-G | 0.444 | 0.811 | 0.945 | 1.36  E-03 | ** | 3.41  E-03 |
| 4-MUG – Telmisartan-G | 0.701 | 0.909 | 0.975 | 4.19  E-05 | *** | 1.57  E-04 |
| 4-MUG – Estradiol-17G | 0.0439 | 0.627 | 0.892 | 3.88  E-02 | * | 5.83  E-02 |
| 4-MUG – Testosterone-G | -0.117 | 0.490 | 0.830 | 1.06  E-01 | ns | 1.23  E-01 |
| PNPG – Indomethacin-G | 0.701 | 0.909 | 0.975 | 4.19  E-05 | *** | 1.57  E-04 |
| PNPG – Telmisartan-G | 0.764 | 0.930 | 0.981 | 1.17  E-05 | *** | 8.78  E-05 |
| PNPG – Estradiol-17G | -0.106 | 0.527 | 0.856 | 9.56  E-02 | ns | 1.19  E-01 |
| PNPG – Testosterone-G | -0.0314 | 0.552 | 0.855 | 6.25  E-02 | ns | 8.52  E-02 |
| Indomethacin-G – Telmisartan-G | 0.306 | 0.748 | 0.925 | 5.12  E-03 | ** | 1.10  E-02 |
| Indomethacin-G – Estradiol-17G | 0.0590 | 0.636 | 0.895 | 3.53  E-02 | * | 5.83  E-02 |
| Indomethacin-G – Testosterone-G | 0.184 | 0.685 | 0.904 | 1.39  E-02 | * | 2.61  E-02 |
| Telmisartan-G – Estradiol-17G | -0.189 | 0.464 | 0.832 | 1.51  E-01 | ns | 1.62  E-01 |
| Telmisartan-G – Testosterone-G | -0.203 | 0.420 | 0.801 | 1.75  E-01 | ns | 1.75  E-01 |
| Estradiol-17G – Testosterone-G | 0.929 | 0.982 | 0.995 | 8.40  E-08 | *** | 1.26  E-06 |


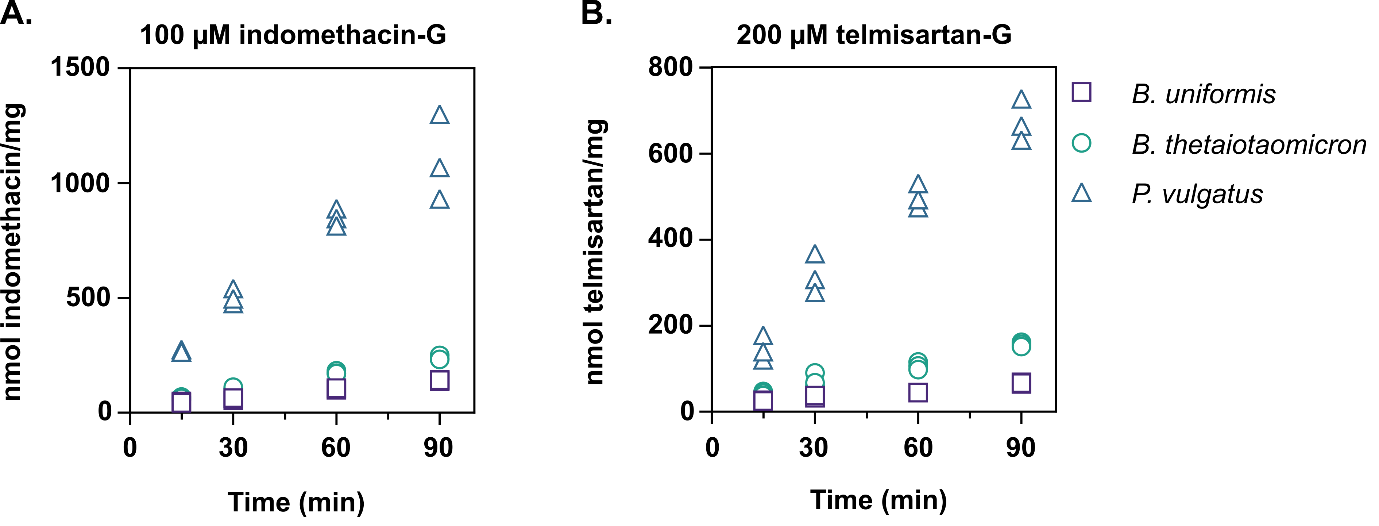


**Supplemental Figure 3.** Deglucuronidation of drug glucuronides by *Bacteroidales* isolates. Indomethacin-G at 100 µM (A) and telmisartan-G at 200 µM (B) were incubated with bacterial lysates produced from cultured isolates of *Bacteroides uniformis*, *B. thetaiotaomicron*, and *Phocaeicola vulgatus*. The reactions were stopped at 15, 30, 60, and 90 min by the addition of ice-cold acetonitrile, after which the formed aglycones were measured with UPLC. Presented are the individual triplicate values originating from a single experiment normalized to the amount of total protein (5 µg) of the reaction.

**Supplemental Table 5.** Correlation of deglucuronidation rates with age and BMI. Spearman correlation was used to correlate age and BMI with the deglucuronidation rates of 200 µM 4-MUG, 500 µM PNPG, and 100 µM indomethacin-G, telmisartan-G, estradiol-17G, and testosterone-G. CI = 95% confidence interval, ρ = Spearman correlation coefficient, sig = significance, ns = not significant, p.adj = Benjamini-Hochberg-adjusted p-value for multiple comparisons.

| **Correlation** | **lower CI** | **ρ** | **upper CI** | **p** | **sig** | **p.adj** |
| --- | --- | --- | --- | --- | --- | --- |
| Age – 4-MUG | -0.493 | 0.113 | 0.645 | 0.727 | ns | 0.850 |
| Age – PNPG | -0.468 | 0.145 | 0.663 | 0.654 | ns | 0.850 |
| Age – Indomethacin-G | -0.562 | 0.018 | 0.586 | 0.957 | ns | 0.957 |
| Age – Telmisartan-G | -0.424 | 0.198 | 0.693 | 0.538 | ns | 0.807 |
| Age – Estradiol-17G | -0.760 | -0.294 | 0.372 | 0.381 | ns | 0.802 |
| Age – Testosterone-G | -0.737 | -0.282 | 0.348 | 0.374 | ns | 0.802 |
| BMI – 4-MUG | -0.732 | -0.273 | 0.357 | 0.391 | ns | 0.802 |
| BMI – PNPG | -0.736 | -0.280 | 0.350 | 0.379 | ns | 0.802 |
| BMI – Indomethacin-G | -0.711 | -0.231 | 0.396 | 0.471 | ns | 0.807 |
| BMI – Telmisartan-G | -0.753 | -0.315 | 0.316 | 0.319 | ns | 0.802 |
| BMI – Estradiol-17G | -0.754 | -0.282 | 0.383 | 0.401 | ns | 0.802 |
| BMI – Testosterone-G | -0.632 | -0.091 | 0.510 | 0.779 | ns | 0.850 |


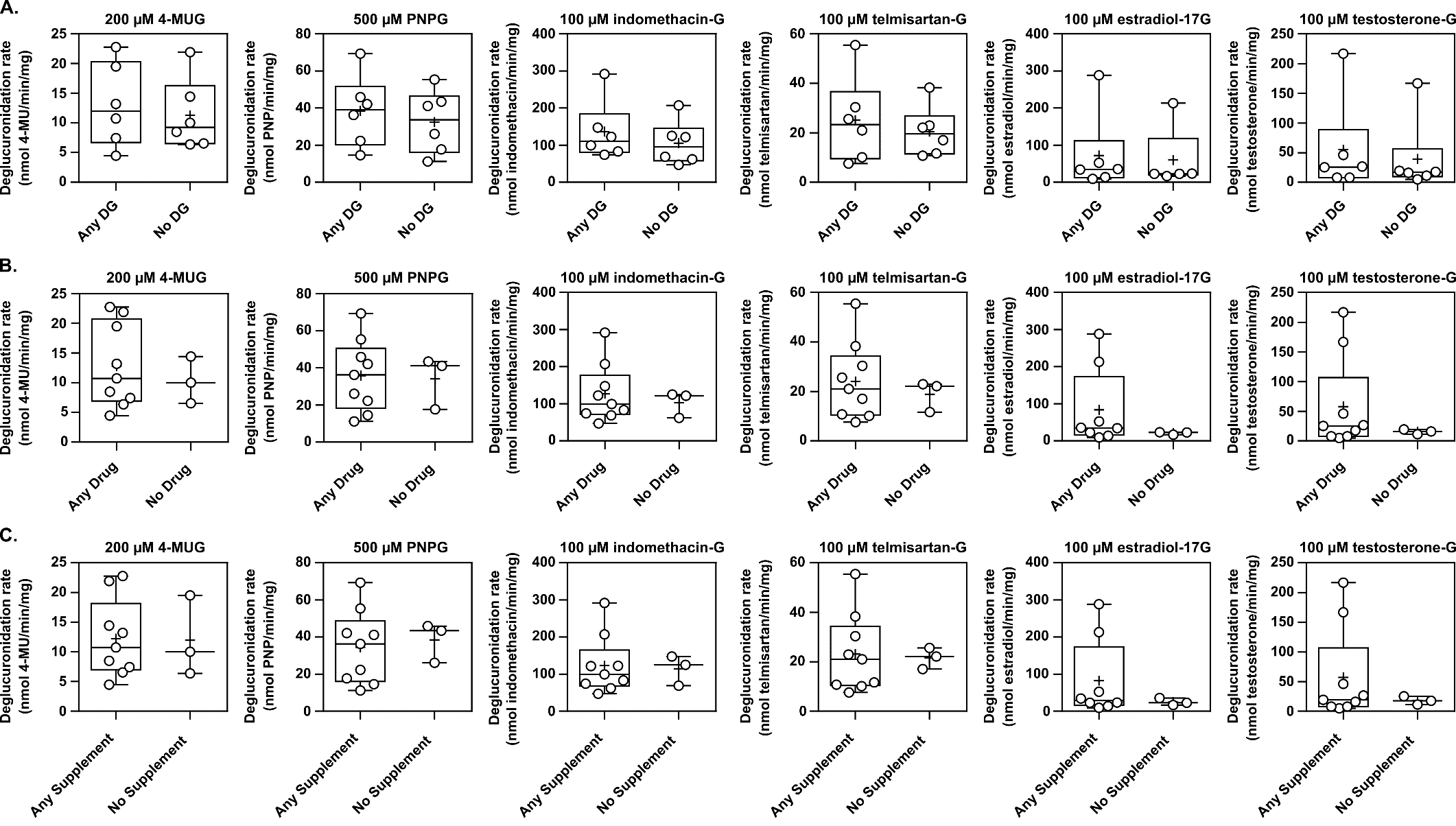


**Supplemental Figure 4.** Association of medical diagnoses and medication and supplement use with deglucuronidation activity. Deglucuronidation rates of 200 µM 4-MUG, 500 µM PNPG, and 100 µM indomethacin-G, telmisartan-G, estradiol-17G, and testosterone-G by samples from donors HD01–HD12 with and without medical diagnoses (DG) (A), reported use of medicines within the past two weeks prior to sample donation (B), and reported use of dietary supplements within the past two weeks prior to sample donation (C). The box extends from the 25^th^ to the 75^th^ percentile with the line representing the median, the cross representing the mean, and the whiskers extending from the minimum to the maximum value.

**
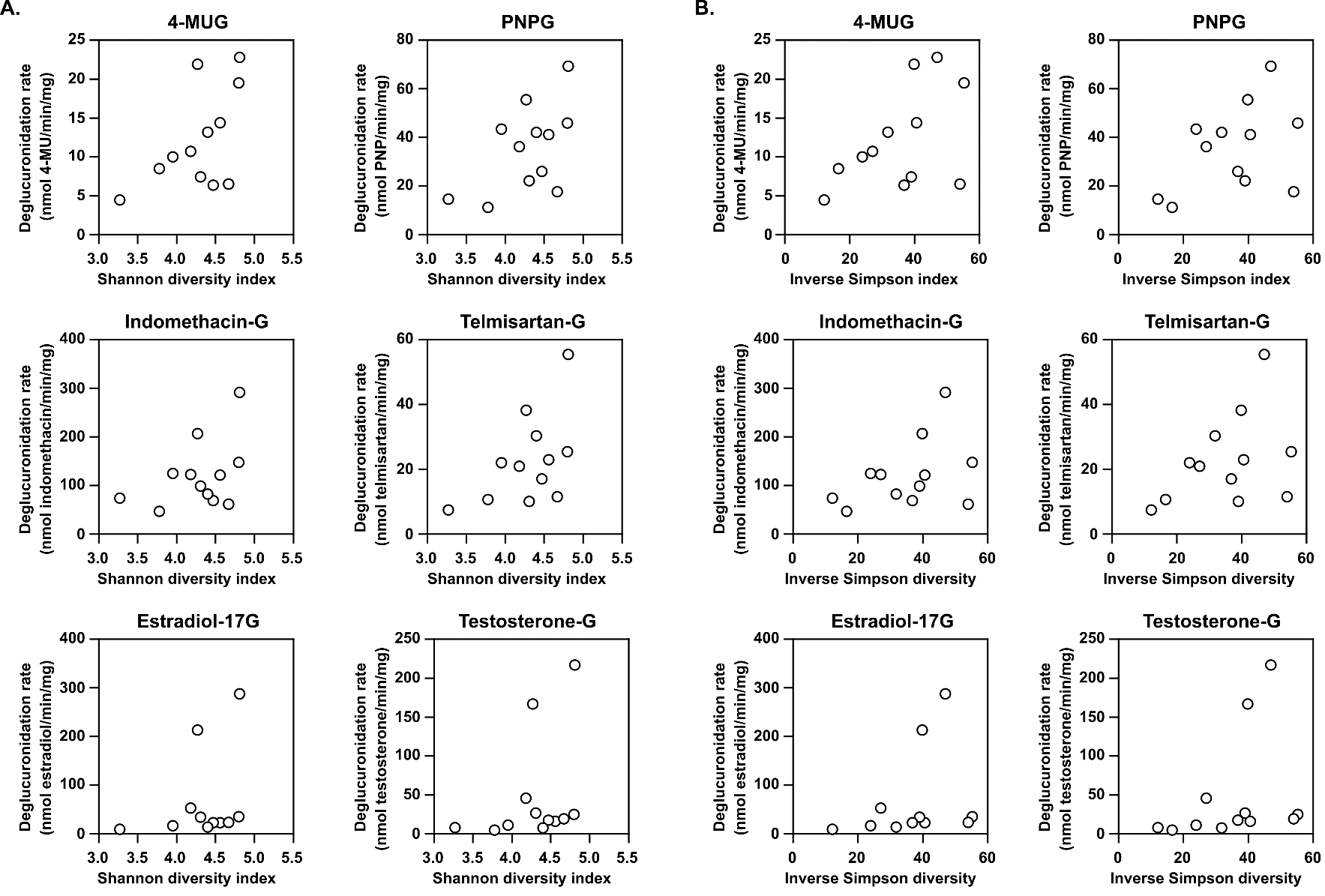
**

**Supplemental Figure 5.** Association of alpha diversity indices with deglucuronidation activity. Shannon (A) and Inverse Simpson (B) diversity indices vs. deglucuronidation rates of 200 µM 4-MUG, 500 µM PNPG, and 100 µM indomethacin-G, telmisartan-G, estradiol-17G, and testosterone-G.

**Supplemental Table 6.** Correlation of deglucuronidation rates with alpha diversity indices. Spearman correlation was used to correlate Shannon and Inverse Simpson diversity indices with deglucuronidation rates of 200 µM 4-MUG, 500 µM PNPG, and 100 µM indomethacin-G, telmisartan-G, estradiol-17G, and testosterone-G. CI = 95% confidence interval, ρ = Spearman correlation coefficient, sig = significance, ns = not significant, * = p < 0.05, p.adj = Benjamini-Hochberg-adjusted p-value for multiple comparisons.

| **Correlation** | **lower CI** | **ρ** | **upper CI** | **p** | **sig** | **p.adj** |
| --- | --- | --- | --- | --- | --- | --- |
| 4-MUG – Shannon | -0.153 | 0.462 | 0.819 | 0.131 | ns | 0.175 |
| PNPG – Shannon | -0.117 | 0.490 | 0.830 | 0.106 | ns | 0.175 |
| Indomethacin-G – Shannon | -0.316 | 0.315 | 0.753 | 0.319 | ns | 0.319 |
| Telmisartan-G – Shannon | -0.0513 | 0.538 | 0.850 | 0.0709 | ns | 0.175 |
| Estradiol-17G – Shannon | -0.232 | 0.427 | 0.818 | 0.190 | ns | 0.227 |
| Testosterone-G – Shannon | -0.135 | 0.476 | 0.824 | 0.118 | ns | 0.175 |
| 4-MUG – Inverse Simpson | -0.0990 | 0.503 | 0.836 | 0.0952 | ns | 0.175 |
| PNPG – Inverse Simpson | -0.117 | 0.490 | 0.830 | 0.106 | ns | 0.175 |
| Indomethacin-G – Inverse Simpson | -0.235 | 0.392 | 0.788 | 0.208 | ns | 0.227 |
| Telmisartan-G – Inverse Simpson | -0.108 | 0.497 | 0.833 | 0.101 | ns | 0.175 |
| Estradiol-17G – Inverse Simpson | -0.0548 | 0.564 | 0.870 | 0.0710 | ns | 0.175 |
| Testosterone-G – Inverse Simpson | 0.0311 | 0.594 | 0.871 | 0.0415 | * | 0.175 |


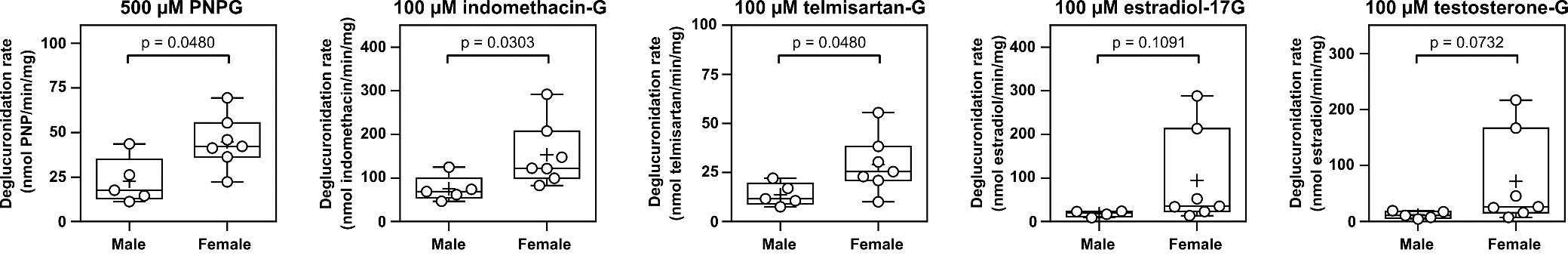


**Supplemental Figure 6.** Sex-dependent deglucuronidation activity. Deglucuronidation rates of 500 µM PNPG and 100 µM indomethacin-G, telmisartan-G, estradiol-17G, and testosterone-G averaged by sex. Mann-Whitney U test was used to test whether the difference in deglucuronidation rates between male and female donors was statistically significant. Reported are the p-values without correction for multiple comparisons, see Supplemental Table 7 for Benjamini-Hochberg-adjusted p-values. The box extends from the 25^th^ to the 75^th^ percentile with the line representing the median, the cross representing the mean, and the whiskers extending from the minimum to the maximum value.

**Supplemental Table 7.** Sex-dependent deglucuronidation activity. Deglucuronidation rates of 500 µM PNPG and 100 µM indomethacin-G, telmisartan-G, estradiol-17G, and testosterone-G averaged by sex, and the Mann-Whitney U test was used to test whether the difference in deglucuronidation rates between female (F) and male (M) donors was statistically significant. Fold = ratio between the mean deglucuronidation rates in females and males, sig = significance, ns = not significant, * = p < 0.05, p.adj = Benjamini-Hochberg-adjusted p-value for multiple comparisons.

| **Substrate** | **Mean, F** | **Mean, M** | **Fold** | **p** | **sig** | **p.adj** |
| --- | --- | --- | --- | --- | --- | --- |
|  | **(nmol/min/mg)** | |  |  |  |  |
| 4-MUG | 15.7 | 6.85 | 2.29 | 0.0101 | * | 0.0606 |
| PNPG | 44.6 | 25.5 | 1.75 | 0.0480 | * | 0.0720 |
| Indomethacin-G | 153 | 82.6 | 1.86 | 0.0303 | * | 0.0720 |
| Telmisartan-G | 29.1 | 14.6 | 2.00 | 0.0480 | * | 0.0720 |
| Estradiol-17G | 94.3 | 18.0 | 5.23 | 0.109 | ns | 0.109 |
| Testosterone-G | 72.1 | 13.9 | 5.19 | 0.0732 | ns | 0.0879 |

**Supplemental Table 8.** Effect of fecal microbiota transplantation on 4-MUG deglucuronidation. Fecal samples from *Clostridioides difficile* patients before and after fecal microbiota transplantation (FMT) were used for 4-MUG deglucuronidation studies. For each patient, the effect of FMT on deglucuronidation activity was measured as the fold of post-FMT deglucuronidation rates compared to the pre-FMT rate. The data were collected from three independent experiments.

| **Sample ID** | **Deglucuronidation rate**  **(nmol 4-MU/min/mg)** | | | **Mean** | **SD** | **Fold** |
| --- | --- | --- | --- | --- | --- | --- |
| P01 pre-FMT | 0.519 | 0.440 | 0.469 | 0.476 | 0.0401 | 1.00 |
| P01 1 month post-FMT | 3.55 | 3.22 | 3.37 | 3.38 | 0.163 | 7.10 |
| P01 1 year post-FMT | 4.58 | 4.24 | 4.33 | 4.38 | 0.180 | 9.20 |
| P02 pre-FMT | 0.131 | 0.0987 | 0.0929 | 0.107 | 0.0204 | 1.00 |
| P02 1 month post-FMT | 1.74 | 1.55 | 1.69 | 1.66 | 0.100 | 15.5 |
| P02 1 year post-FMT | 3.25 | 2.98 | 3.15 | 3.13 | 0.140 | 29.1 |
| P03 pre-FMT | 0.654 | 0.556 | 0.630 | 0.613 | 0.0515 | 1.00 |
| P03 1 month post-FMT | 6.20 | 5.07 | 5.46 | 5.58 | 0.577 | 9.09 |
| P03 1 year post-FMT | 9.49 | 7.90 | 9.03 | 8.80 | 0.817 | 14.4 |
| P04 pre-FMT | 3.20 | 2.60 | 2.76 | 2.85 | 0.308 | 1.00 |
| P04 1 month post-FMT | 1.40 | 1.16 | 1.23 | 1.26 | 0.125 | 0.44 |
| P04 1 year post-FMT | 9.19 | 7.77 | 8.57 | 8.51 | 0.708 | 2.98 |
| P05 pre-FMT | 1.17 | 1.03 | 1.10 | 1.10 | 0.0724 | 1.00 |
| P05 1 month post-FMT | 6.94 | 5.93 | 6.51 | 6.46 | 0.508 | 5.88 |
| P05 1 year post-FMT | 3.66 | 3.33 | 3.56 | 3.52 | 0.166 | 3.20 |
| P06 pre-FMT | 3.71 | 3.32 | 3.56 | 3.53 | 0.194 | 1.00 |
| P06 1 month post-FMT | 9.03 | 6.77 | 8.40 | 8.07 | 1.16 | 2.29 |
| P06 1 year post-FMT | 4.17 | 3.77 | 4.10 | 4.01 | 0.214 | 1.14 |
| P07 pre-FMT | 3.36 | 2.80 | 3.13 | 3.10 | 0.279 | 1.00 |
| P07 1 month post-FMT | 3.16 | 2.76 | 3.02 | 2.98 | 0.205 | 0.96 |
| P07 1 year post-FMT | 4.13 | 3.40 | 3.81 | 3.78 | 0.366 | 1.22 |
